# Postponing colonoscopy for 6 months in high‐risk population increases colorectal cancer detection in China

**DOI:** 10.1002/cam4.5850

**Published:** 2023-03-23

**Authors:** Mingqing Zhang, Yongdan Zhang, Wen Zhang, Lizhong Zhao, Haoren Jing, Xiaojing Wu, Lu Guo, Haixiang Zhang, Yong Zhang, Siwei Zhu, Shiwu Zhang, Xipeng Zhang

**Affiliations:** ^1^ Nankai University School of Medicine Nankai University Tianjin China; ^2^ Department of Colorectal Surgery Tianjin Union Medical Center Tianjin China; ^3^ Tianjin Institute of Coloproctology Tianjin China; ^4^ The Institute of Translational Medicine Tianjin Union Medical Center of Nankai University Tianjin China; ^5^ Center for Applied Mathematics Tianjin University Tianjin China; ^6^ Department of Pathology Tianjin Union Medical Center Tianjin China

**Keywords:** cancer prevention, colonoscopy, colorectal cancer, colorectal cancer screening

## Abstract

**Background and Aims:**

Colonoscopy is an important colorectal cancer (CRC) screening modality; however, not all high‐risk groups identified by fecal immunochemical test (FIT) and/or high‐risk factor questionnaire (HRFQ) undergo colonoscopy in time. The impact of delays in colonoscopy on CRC detection among high‐risk populations remains poorly understood, warranting further clarification.

**Methods:**

A retrospective study was conducted among CRC high‐risk population identified by Tianjin CRC screening program. According to the colonoscopy results after HRFQ and FIT, patients were classified into CRC, advanced adenoma, non‐advanced adenoma, and normal groups. The time interval between CRC screening and colonoscopy was investigated and its relationship with colonoscopy results. Logistic regression was performed to explore the risk factors of CRC detection.

**Results:**

Among the high‐risk population without a history of CRC or polyps, 49,810 underwent HRFQ, FIT, and colonoscopy, and a time interval of fewer than 6 months was found for 79.56% of patients (*n* = 39,630). People with positive FIT were more likely to undergo colonoscopy within 6 months, and detection rates of CRC and/or advanced adenoma were positively related to time intervals. Similar results were found in people with a negative FIT but positive HRFQ. A time interval longer than 6 months was a significant predictor of CRC detection in high‐risk populations.

**Conclusion:**

For high‐risk people identified by CRC screening, especially those with a positive FIT, a time interval of 6 months was associated with an increased probability of CRC detection. Our findings emphasize that populations at high risk should undergo colonoscopy at least within 6 months.

## Graphical abstract

Delayed colonoscopy is associated with increased colorectal cancer detection in people at high risk for colorectal cancer.

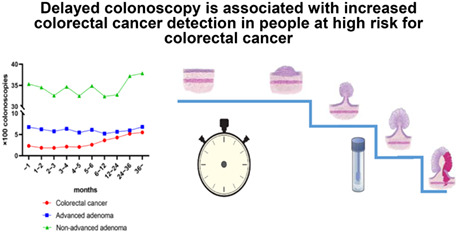

## 1 INTRODUCTION

Colorectal cancer (CRC) is one of the most common malignancies with an increasing incidence worldwide.^1^ CRC screening enable early detection of advanced adenomas and other lesions to reduce the incidence and mortality of CRC.^2^, ^3^ Commonly used screening strategies among high‐risk populations are based on risk stratification and/or fecal immunochemical test (FIT)/fecal occult blood test (FOBT), followed by further colonoscopy.^4^, ^5^, ^6^ However, the compliance and timeliness of colonoscopy are unsatisfactory due to the need for bowel preparation, the invasive nature, and psychological factors.^7^ Given that part of the high‐risk population cannot undergo colonoscopy in time, the time interval between CRC screening and colonoscopy may undermine the power of CRC screening.^8^

No consensus has been reached on the optimal interval time probably due to heterogeneity in regions and ethnicities.^9^, ^10^, ^11^, ^12^, ^13^, ^14^ This is also reflected in different CRC screening guidelines, where the time requirements for a colonoscopy after a positive FIT are broad and diverse. In addition, there is a lack of adequate and clear guidance on the interval time among high‐risk stratification populations with negative FIT.^15^

Since 2012, CRC screening for asymptomatic people aged 40–74 years has been initiated in Tianjin.^16^ As of 2020, a total of 49,810 people in the high‐risk group underwent high‐risk factor questionnaire (HRFQ), FIT, and colonoscopy, with colonoscopy delayed more than 6 months in more than 20% of cases. In this retrospective study, we focused on high‐risk populations in the CRC screening process who underwent a colonoscopy to investigate the effect of prolonged time interval on the detection rate of CRC, advanced adenoma and non‐advanced adenoma. Our results shed light on the optimal time for colonoscopy in CRC high‐risk populations.

## 2 METHODS

### 2.1 Study population

The CRC screening program was a free‐of‐charge scheme established for the asymptomatic population aged 40–74 in a 3‐year cycle between 2012 and 2020. The main screening population was residents aged 60–74 years in 2012, 50–60 years in 2013, and 40–50 years in 2014. The same protocol was carried out from 2015 to 2017 and from 2018 to 2020. After providing informed consent, all participants answered a HRFQ followed by FIT. Patients that met one of the following conditions were attributed to the positive HRFQ: (a) patients with a first‐degree relative with CRC; (b) a history of cancer or intestinal polyps; (c) patients with two or more of the following: a history of chronic constipation, a history of chronic diarrhea, a history of bloody mucous stools, adverse life events (e.g., divorce, death of a close relative, etc.) a history of chronic appendicitis or appendectomy, a history of chronic cholecystitis or gallstones. High‐risk population were defined as positive HRFQ or positive FIT. These high‐risk population were then recommended to undergo a free colonoscopy, and for those at risk who decide to have a colonoscopy, the procedure is usually completed within a month. Those who did not undergo the colonoscopy were reminded by telephone annually until completion. No deadlines were set for undergoing the colonoscopy.

### 2.2 Data collection

Data were collected and entered into a database by experienced physicians. We analyzed data of all high‐risk individuals that underwent colonoscopy from 2012 to 2020, including demographics (e.g., age, gender, education), time and results of HRFQ, FIT, and colonoscopy. Abnormal findings on colonoscopy were classified as CRC, advanced adenoma (i.e., patients with adenoma of diameter ≥1 cm, villous adenomas with at least 25% of villous components, or adenomas with high‐grade dysplasia, serrated adenoma/sessile serrated polyp ≥10 mm or with high‐grade dysplasia and traditional serrated adenomas), and non‐advanced adenoma. Patients with benign diseases such as lipoma, diverticulum, and colitis were incorporated into the normal group for analysis. Patients were excluded from the study based on the following criteria: (a) a history of colonic polyps or CRC; (b) missing data; (c) unable to complete colonoscopy due to poor bowel preparation during the whole screening program (if poor bowel preparation occurred in the first colonoscopy, the second colonoscopy results were used). Unlike our previous article,^17^ only data from the first time identified high‐risk population were considered for subjects who participated in multiple CRC screenings during the study period. In addition, we updated the pathological biopsy results of screening colonoscopy based on the patient's postoperative pathological findings if the information was available. Information about CRC cancer stage according to the TNM stages specified by the 8th edition of the American Joint Committee on Cancer was extracted from clinical records and histopathology reports in Tianjin Union Medical Center.

### 2.3 Outcomes

Calculated based on the time interval were the following indicators: (1) percentage distribution of colonoscopies; (2) detection of CRCs by TNM stages (% colonoscopies); (3) detection of advanced and non‐advanced adenoma (% colonoscopies). We divided the population into FIT‐negative or ‐positive groups and analyzed the changes in the detection rate of CRC at different intervals.

### 2.4 Exposure

The primary exposure (predictor) variable was the time interval between CRC screenings and colonoscopy. Time intervals were divided into 10 groups: within 1 month (≤30 days), 1–2 months (31–60 days), 2–3 months (60–90 days), 3–4 months (91–120 days), 4–5 months (121–150 days), 5–6 months (151–180 days), 6–12 months (181–360 days), 13–24 months (361–720 days), 25–36 months (721–1080 days), and more than 36 months (≥1081 days).

### 2.5 Statistical analysis

A descriptive analysis was conducted to characterize demographics and clinical factors in the study cohort and CRC outcome rates based on the time to colonoscopy. We used logistic regression models to analyze the relationship between time to colonoscopy and outcome indicators, for example, CRC. Differences in CRC, advanced adenoma and non‐advanced adenoma between groups were assessed by univariate analysis. In multivariate logistic regression analysis, the significant factors from univariate analysis were further used to identify independent risk factors for advanced adenoma, CRC, and non‐advanced adenoma. All multivariate models were adjusted for age, gender, education, occupation, residence, FIT, history of chronic diarrhea, history of chronic constipation, history of bloody mucous stools, history of chronic appendicitis or appendectomy, history of chronic cholecystitis or gallstones, adverse life events, history of cancer, and history of CRC in a first‐degree relative at the time of FIT. The corresponding odds ratios (ORs) and confidence intervals (CIs) with 95% confidence were calculated for each independent risk factor. We used completion of colonoscopy within 1 month as our reference group. In sensitivity analyses, the reference group was redefined to include patients who had their colonoscopies within 8–30 days (excluding whose time intervals <7 days and who were unable to determine the exact time interval), <3 months and <6 months after identified as high risk; including members who had a history of colonic polyps prior to CRC screening; and then adding an exposure category of 1–7 days when 8–30 days was the reference group. Numerical data were described by the number of cases or constituent ratio. A value of *p* < 0.05 was statistically significant. All statistical analyses were conducted using R software, Version 4.1.2.

## 3 RESULTS

### 3.1 Characteristics of the study population

The CRC screening program identified that a total of 72,269 asymptomatic people aged 40–74 years were at high risk, and 49,810 cases were included in our study (Figure S1). The median age was 60 years, with 22,655 (45.48%) males and 27,155 (54.52%) females; the proportion of people aged 60–69 years was the highest (*n* = 22,307, 44.78%). There were 33,469 (67.19%) positive FIT, and 21,915 (44.00%) were assessed as high risk by the questionnaire‐based risk assessment. A total of 1328 cases (2.67%) of CRC, 3150 cases (6.32%) of advanced adenoma, 17,297 cases (34.73%) of non‐advanced adenoma, and 28,035 cases (56.28%) of normal group were detected. Among the CRCs, there were 56 (4.22%) cases of stage 0, 255 (19.20%) cases of stage I, 328 (24.70%) cases of stage II, 296 (22.29%) cases of advanced CRC of stage III, and 55 (4.14%) cases of stage IV. The interval was less than 30 days for 19,631 (39.41%) participants, less than 6 months for 39,630 (79.56%) participants and more than 6 months for 10,180 (20.44%) participants (Table 1, Figure S2).

**TABLE 1:** Characteristics and CRC outcomes in high‐risk population undergoing colonoscopy.

Time to colonoscopy after FIT, months	Total, *n* (%)	1	2	3	4	5	6	7–12	13–24	25–36	37~
Gender											
Male	22,655 (45.48)	9172 (46.72)	4011 (45.8)	2563 (47.05)	1341 (44)	813 (47.8)	481 (45.98)	1059 (43.17)	996 (41.6)	761 (43.76)	1458 (40.57)
Female	27,155 (54.52)	10,459 (53.28)	4746 (54.2)	2884 (52.95)	1707 (56)	888 (52.2)	565 (54.02)	1394 (56.83)	1398 (58.4)	978 (56.24)	2136 (59.43)
Median age, years	60	59	60	61	62	62	62	62	60	61	60
Age											
40–49	6102 (12.25)	3191 (16.25)	1112 (12.7)	463 (8.5)	264 (8.66)	133 (7.82)	99 (9.46)	188 (7.66)	177 (7.39)	139 (7.99)	336 (9.35)
50–59	16,796 (33.72)	7325 (37.31)	2856 (32.61)	1683 (30.9)	730 (23.95)	472 (27.75)	245 (23.42)	727 (29.64)	871 (36.38)	543 (31.22)	1344 (37.4)
60–69	22,307 (44.78)	7569 (38.56)	3931 (44.89)	2692 (49.42)	1680 (55.12)	892 (52.44)	566 (54.11)	1264 (51.53)	1129 (47.16)	886 (50.95)	1698 (47.25)
70‐	4605 (9.25)	1546 (7.88)	858 (9.8)	609 (11.18)	374 (12.27)	204 (11.99)	136 (13)	274 (11.17)	217 (9.06)	171 (9.83)	216 (6.01)
Education											
Elementary school/above	37,648 (75.58)	15,114 (76.99)	6355 (72.57)	3880 (71.23)	2188 (71.78)	1249 (73.43)	757 (72.37)	1965 (80.11)	1881 (78.57)	1397 (80.33)	2862 (79.63)
Elementary school/below	12,162 (24.42)	4517 (23.01)	2402 (27.43)	1567 (28.77)	860 (28.22)	452 (26.57)	289 (27.63)	488 (19.89)	513 (21.43)	342 (19.67)	732 (20.37)
Occupation											
Mental work	14,418 (28.95)	5521 (28.12)	2485 (28.38)	1517 (27.85)	896 (29.4)	525 (30.86)	332 (31.74)	773 (31.51)	744 (31.08)	511 (29.38)	1114 (31)
Manual work	35,392 (71.05)	14,110 (71.88)	6272 (71.62)	3930 (72.15)	2152 (70.6)	1176 (69.14)	714 (68.26)	1680 (68.49)	1650 (68.92)	1228 (70.62)	2480 (69)
Residential area											
Central urban	18,520 (37.18)	6969 (35.5)	3008 (34.35)	1622 (29.78)	999 (32.78)	595 (34.98)	433 (41.4)	1227 (50.02)	1066 (44.53)	821 (47.21)	1780 (49.53)
Agriculture‐related areas	31,290 (62.82)	12,662 (64.5)	5749 (65.65)	3825 (70.22)	2049 (67.22)	1106 (65.02)	613 (58.6)	1226 (49.98)	1328 (55.47)	918 (52.79)	1814 (50.47)
HRFQ											
Positive	21,915 (44)	6736 (34.31)	3482 (39.76)	2359 (43.31)	1502 (49.28)	836 (49.15)	498 (47.61)	1403 (57.2)	1445 (60.36)	1119 (64.35)	2535 (70.53)
Negative	27,895 (56)	12,895 (65.69)	5275 (60.24)	3088 (56.69)	1546 (50.72)	865 (50.85)	548 (52.39)	1050 (42.8)	949 (39.64)	620 (35.65)	1059 (29.47)
FIT											
Positive	33,469 (67.19)	15,547 (79.2)	6269 (71.59)	3713 (68.17)	1867 (61.25)	1022 (60.08)	640 (61.19)	1272 (51.85)	1107 (46.24)	723 (41.58)	1309 (36.42)
Negative	16,341 (32.81)	4084 (20.8)	2488 (28.41)	1734 (31.83)	1181 (38.75)	679 (39.92)	406 (38.81)	1181 (48.15)	1287 (53.76)	1016 (58.42)	2285 (63.58)
History of chronic diarrhea											
Yes	11,320 (22.73)	4274 (21.77)	1968 (22.47)	1388 (25.48)	662 (21.72)	370 (21.75)	233 (22.28)	557 (22.71)	542 (22.64)	399 (22.94)	927 (25.79)
No	38,490 (77.27)	15,357 (78.23)	6789 (77.53)	4059 (74.52)	2386 (78.28)	1331 (78.25)	813 (77.72)	1896 (77.29)	1852 (77.36)	1340 (77.06)	2667 (74.21)
History of chronic constipation											
Yes	11,568 (23.22)	4107 (20.92)	1933 (22.07)	1164 (21.37)	749 (24.57)	410 (24.1)	257 (24.57)	637 (25.97)	667 (27.86)	539 (30.99)	1105 (30.75)
No	38,242 (76.78)	15,524 (79.08)	6824 (77.93)	4283 (78.63)	2299 (75.43)	1291 (75.9)	789 (75.43)	1816 (74.03)	1727 (72.14)	1200 (69.01)	2489 (69.25)
History of bloody mucous stools											
Yes	8280 (16.62)	3384 (17.24)	1349 (15.4)	861 (15.81)	493 (16.17)	314 (18.46)	139 (13.29)	444 (18.1)	368 (15.37)	274 (15.76)	654 (18.2)
No	41,530 (83.38)	16,247 (82.76)	7408 (84.6)	4586 (84.19)	2555 (83.83)	1387 (81.54)	907 (86.71)	2009 (81.9)	2026 (84.63)	1465 (84.24)	2940 (81.8)
History of chronic appendicitis or appendectomy											
Yes	4920 (9.88)	1413 (7.2)	788 (9)	533 (9.79)	351 (11.52)	197 (11.58)	110 (10.52)	336 (13.7)	342 (14.29)	260 (14.95)	590 (16.42)
No	44,890 (90.12)	18,218 (92.8)	7969 (91)	4914 (90.21)	2697 (88.48)	1504 (88.42)	936 (89.48)	2117 (86.3)	2052 (85.71)	1479 (85.05)	3004 (83.58)
History of chronic cholecystitis or gallstones											
Yes	4571 (9.18)	1284 (6.54)	719 (8.21)	467 (8.57)	301 (9.88)	175 (10.29)	108 (10.33)	306 (12.47)	341 (14.24)	264 (15.18)	606 (16.86)
No	45,239 (90.82)	18,347 (93.46)	8038 (91.79)	4980 (91.43)	2747 (90.12)	1526 (89.71)	938 (89.67)	2147 (87.53)	2053 (85.76)	1475 (84.82)	2988 (83.14)
Adverse life events											
Yes	5859 (11.76)	1472 (7.5)	890 (10.16)	778 (14.28)	527 (17.29)	222 (13.05)	125 (11.95)	378 (15.41)	388 (16.21)	310 (17.83)	769 (21.4)
No	43,951 (88.24)	18,159 (92.5)	7867 (89.84)	4669 (85.72)	2521 (82.71)	1479 (86.95)	921 (88.05)	2075 (84.59)	2006 (83.79)	1429 (82.17)	2825 (78.6)
History of cancer											
Yes	1621 (3.25)	402 (2.05)	260 (2.97)	132 (2.42)	140 (4.59)	74 (4.35)	46 (4.4)	119 (4.85)	123 (5.14)	99 (5.69)	226 (6.29)
No	48,189 (96.75)	19,229 (97.95)	8497 (97.03)	5315 (97.58)	2908 (95.41)	1627 (95.65)	1000 (95.6)	2334 (95.15)	2271 (94.86)	1640 (94.31)	3368 (93.71)
History of CRC in a first‐degree relative											
Yes	5141 (10.32)	1534 (7.81)	754 (8.61)	483 (8.87)	339 (11.12)	212 (12.46)	145 (13.86)	387 (15.78)	390 (16.29)	290 (16.68)	607 (16.89)
No	44,669 (89.68)	18,097 (92.19)	8003 (91.39)	4964 (91.13)	2709 (88.88)	1489 (87.54)	901 (86.14)	2066 (84.22)	2004 (83.71)	1449 (83.32)	2987 (83.11)
Diagnosis											
CRC	1328 (2.67)	456 (2.32)	164 (1.87)	101 (1.85)	65 (2.13)	35 (2.06)	27 (2.58)	89 (3.63)	103 (4.3)	90 (5.18)	198 (5.51)
Advanced adenoma	3150 (6.32)	1328 (6.76)	546 (6.24)	313 (5.75)	194 (6.36)	93 (5.47)	64 (6.12)	128 (5.22)	135 (5.64)	104 (5.98)	245 (6.82)
Non‐advanced adenoma	17,297 (34.73)	6933 (35.32)	3025 (34.54)	1776 (32.61)	1057 (34.68)	553 (32.51)	365 (34.89)	795 (32.41)	785 (32.79)	647 (37.21)	1361 (37.87)
Normal	28,035 (56.28)	10,914 (55.6)	5022 (57.35)	3257 (59.79)	1732 (56.82)	1020 (59.96)	590 (56.41)	1441 (58.74)	1371 (57.27)	898 (51.64)	1790 (49.81)
CRC stage											
0	56 (4.22)	25 (5.48)	26 (5.63)	27 (5.77)	28 (5.91)	29 (6.04)	30 (6.17)	31 (6.30)	32 (6.43)	33 (6.55)	34 (6.67)
I	255 (19.20)	96 (21.05)	97 (21.00)	98 (20.94)	99 (20.89)	100 (20.83)	101 (20.78)	102 (20.73)	103 (20.68)	104 (20.63)	105 (20.59)
II	328 (24.70)	110 (24.12)	111 (24.03)	112 (23.93)	113 (23.84)	114 (23.75)	115 (23.66)	116 (23.58)	117 (23.49)	118 (23.41)	119 (23.33)
III	296 (22.29)	86 (18.86)	87 (18.83)	88 (18.80)	89 (18.78)	90 (18.75)	91 (18.72)	92 (18.70)	93 (18.67)	94 (18.65)	95 (18.63)
IV	55 (4.14)	14 (3.07)	15 (3.25)	16 (3.42)	17 (3.59)	18 (3.75)	19 (3.91)	20 (4.07)	21 (4.22)	22 (4.37)	23 (4.51)
Unknown	338 (25.45)	125 (27.41)	126 (27.27)	127 (27.14)	128 (27.00)	129 (26.88)	130 (26.75)	131 (26.63)	132 (26.51)	133 (26.39)	134 (26.27)
Advanced‐stage CRC	351 (35.45)	100 (30.21)	37 (32.46)	21 (29.58)	16 (33.33)	5 (26.32)	8 (42.11)	28 (43.08)	46 (50.55)	24 (34.29)	66 (40.74)
Early‐stage CRC	639 (64.55)	231 (69.79)	77 (67.54)	50 (70.42)	32 (66.67)	14 (73.68)	11 (57.89)	37 (56.92)	45 (49.45)	46 (65.71)	96 (59.26)
Total	49,810	19,631	8757	5447	3048	1701	1046	2453	2394	1739	3594

The HRFQ mainly covered related factors such as symptoms and personal history.^18^ A slight increase in the proportion of people with a positive history of relevant symptoms or personal history over time suggested that the presence of these conditions did not prompt patients to undergo colonoscopy as early as possible. However, the proportion of people with positive FIT decreased significantly over time, consistent with the analysis of colonoscopy compliance,^16^ proving the positive impact of FIT on encouraging timely colonoscopy in high‐risk population.

### 3.2 Time interval and incidence of CRC outcomes

**FIGURE 1:**
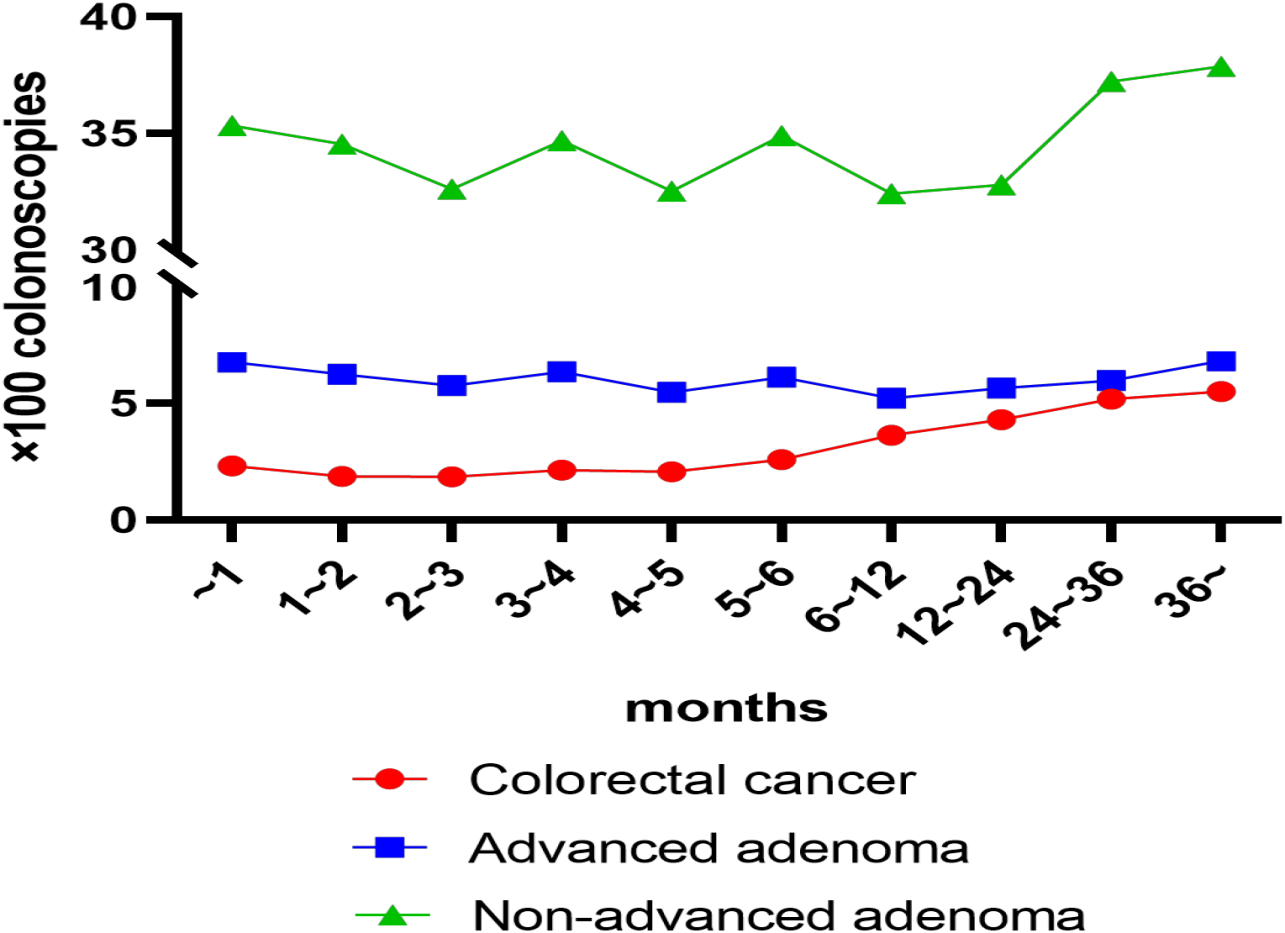
Detection of colorectal cancer, advanced adenoma and non‐advanced adenoma in patients awaiting colonoscopy after identified as high‐risk population.

**FIGURE 2:**
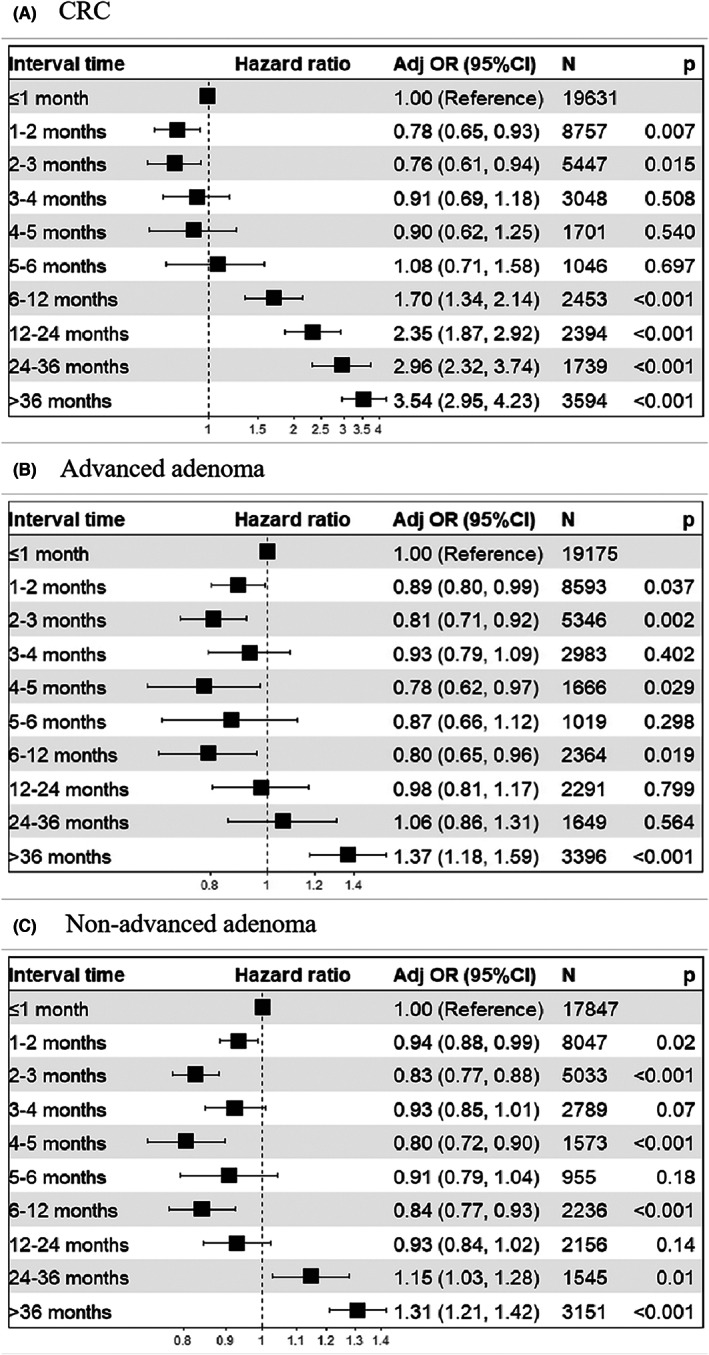
Time to colonoscopy after identified as high‐risk population and adjusted incidence of CRC (A), advanced adenoma (B), non‐advanced adenoma (C).

Colonoscopy results were tabulated by group according to the time intervals as shown in Table 1 (Figure 1). Compared with people that underwent colonoscopy within 1 month, the incidence of CRC was slightly decreased in those who underwent colonoscopy within 2–6 months. However, from 7 to 12 months, the incidence of CRC significantly increased (OR 1.70, 95% CI 1.34–2.14) (Figure 2A, Table S1), and the incidence was heightened with increased interval duration. In our study, no increase in non‐advanced adenoma or advanced adenoma was observed with an interval over 24 months. After an interval of more than 36 months, the incidence of advanced adenomas detection was significantly increased (OR 1.37, 95% CI 1.18–1.59) (Figure 2B, Table S2). Moreover, the incidence of non‐advanced adenoma detection was significantly increased with an interval of more than 24 months (OR 1.15, 95% CI 1.03–1.28) (Figure 2C, Table S3).

**FIGURE 3:**
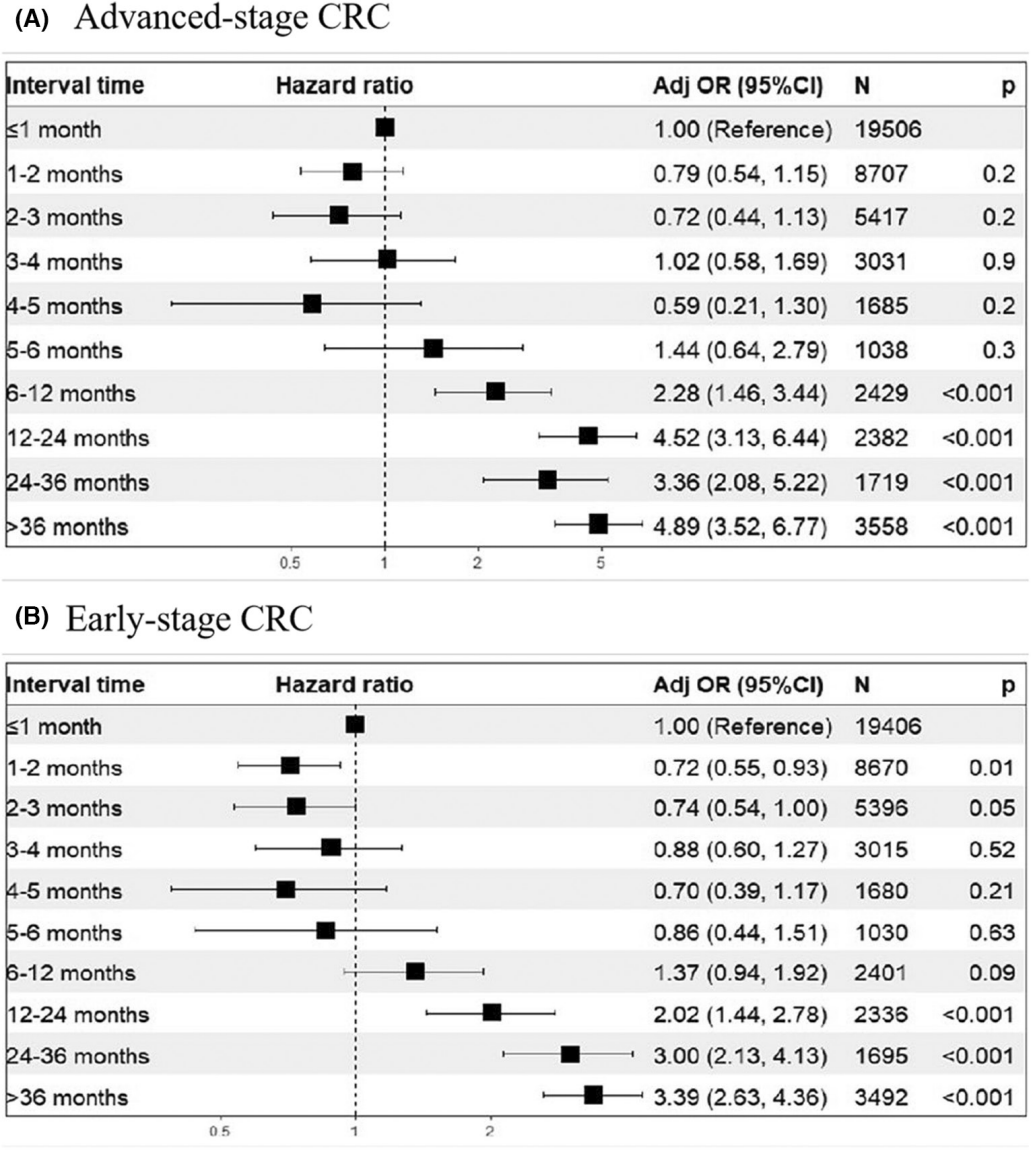
Time to colonoscopy after identified as high‐risk population and adjusted incidence of advanced‐stage colorectal cancer (A), early‐stage CRC (B). CI, confidence interval. The model was adjusted for age, gender, education, occupation, residence, FIT, history of chronic diarrhea, history of chronic constipation, history of bloody mucous stools, history of chronic appendicitis or appendectomy, history of chronic cholecystitis or gallstones, adverse life events, history of cancer, and a first‐degree relative with CRC. A full analysis set was used for the CRC model, while 1328 CRC cases were excluded in the advanced adenoma model, and an additional 3150 advanced adenoma cases were excluded in the non‐advanced adenoma model.

We further analyzed the clinical stages of 990 (74.55%) CRC cases, including 56 (4.22%), 255 (19.20%), and 328 (24.70%) cases at stages 0, I, and II, respectively. The proportion of advanced cases (i.e., stages III and IV) increased in patients with intervals longer than 6 months (OR 2.28, 95% CI 1.46–3.44) (Figure 3A, Table S4). In contrast, the detection rate of early‐stage CRC increased when the interval was more than 12 months (OR 2.02, 1.44–2.78) (Figure 3B, Table S5).

### 3.3 Time interval and incidence of CRC findings among those with FIT results

**FIGURE 4:**
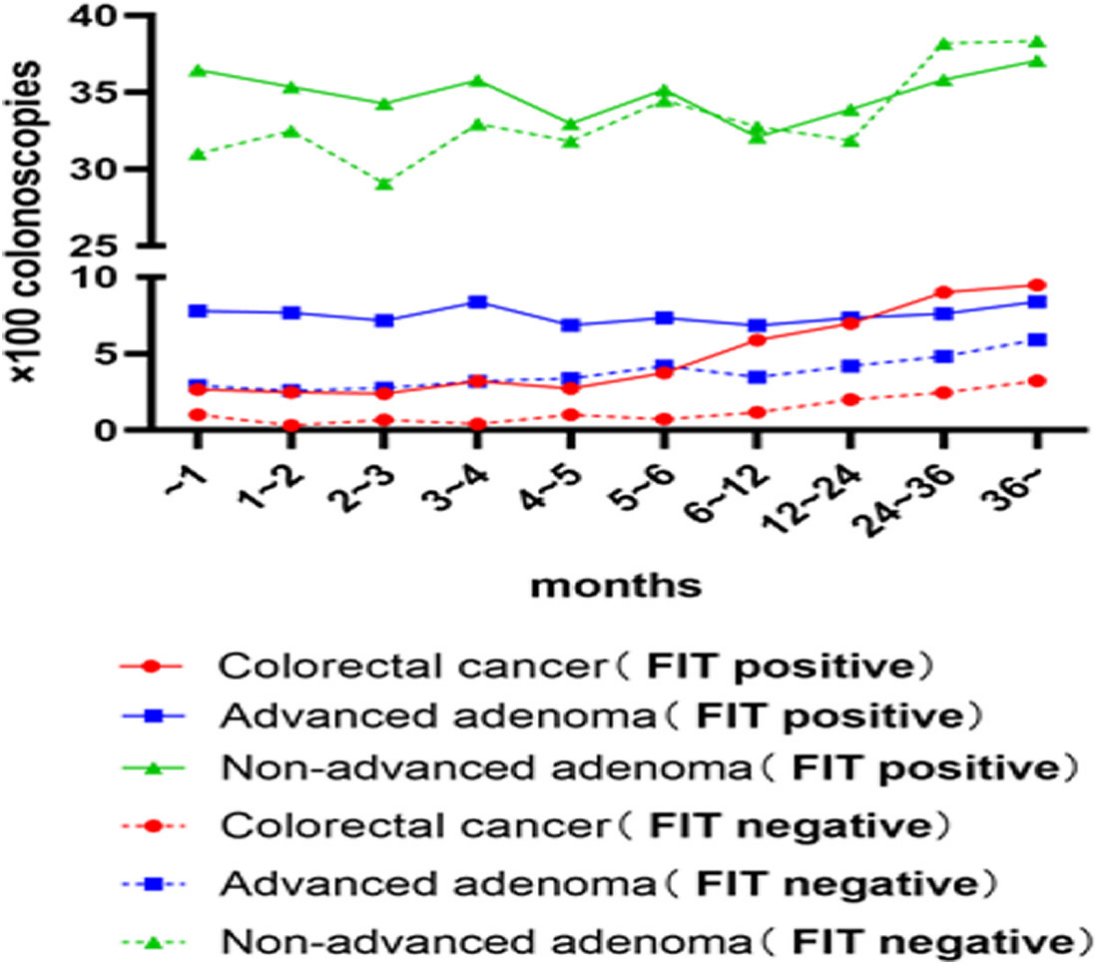
Detection of colorectal cancer, advanced adenoma and non‐advanced adenoma in positive (solid line) and negative (dotted line) fecal immunochemical test patients, according to the time interval for colonoscopy after identified as high‐risk population.

**FIGURE 5:**
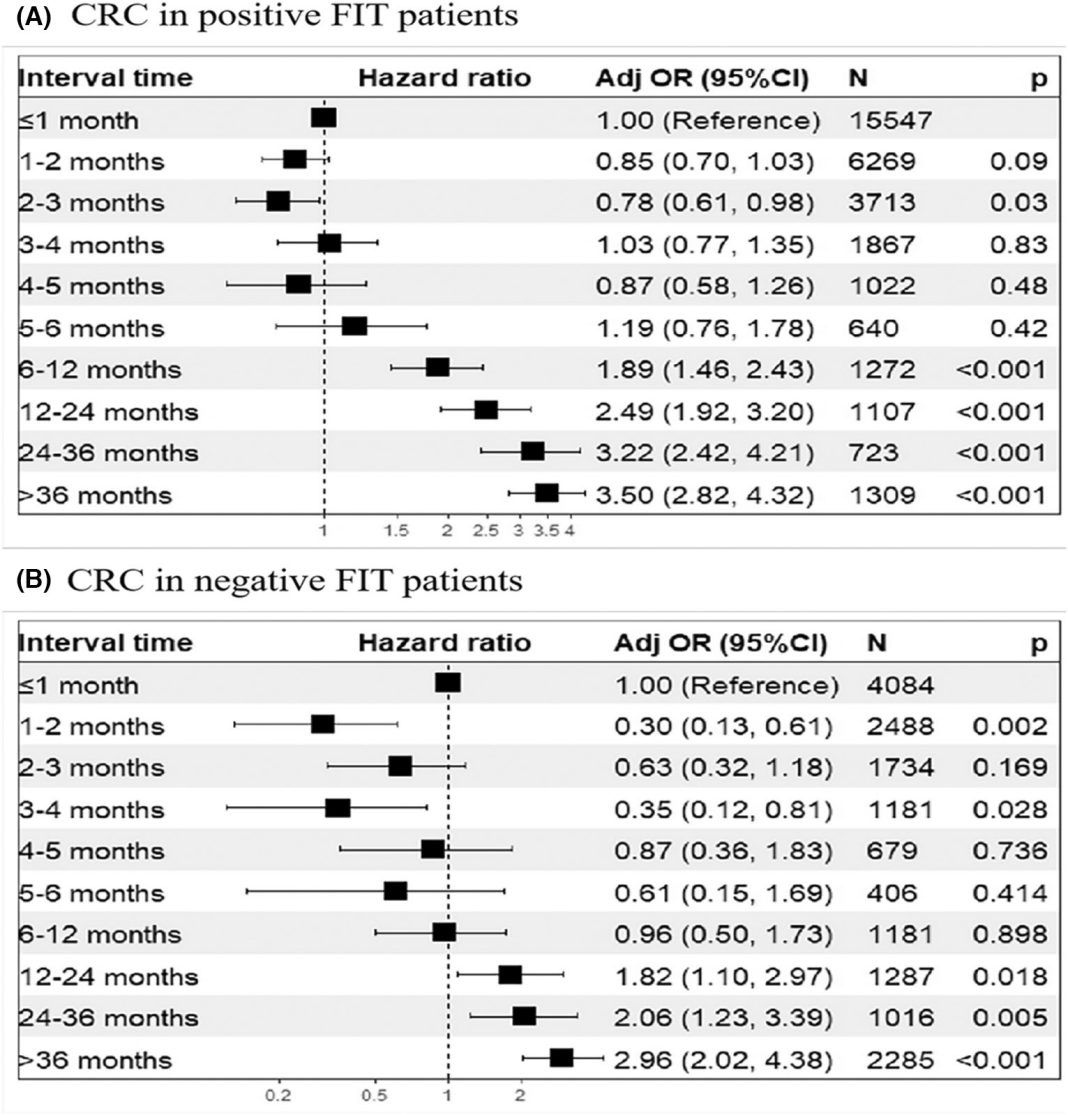
Time to colonoscopy after a positive fecal immunochemical test (FIT) (A), negative FIT (B) and the adjusted incidence of colorectal cancer.

A previous study demonstrated that delayed colonoscopy among people with positive FIT could lead to CRC, which might be related to fecal hemoglobin levels.^12^, ^19^ Therefore, we conducted a separate analysis on high‐risk populations with positive and negative FIT results. We found that in people with a positive FIT, the detection rate of CRC was higher in participants who had delayed colonoscopy for >6 months than in those who underwent colonoscopy within 6 months, and the detection rate gradually increased with the interval (OR_min_ 1.89, 95% CI 1.46–2.43) (Figures 4 and 5A, Table S6). The same conclusion was reached in the negative HRFQ population (Table S9, Figure S3B), and the same trend was observed in the population that was positive for both the HRFQ and the FIT (Table S10, Figure S3C), but the detection of CRC did not increase significantly until after 36 months.

Prior evidence has shown a relatively low risk of CRC detection in people with negative FIT^20^, ^21^. We found that the risk of CRC in high‐risk people with a negative FIT was also positively correlated to the time interval (i.e., greater than 12 months) (OR 1.82, 95% CI 1.10–2.97) (Figures 4 and 5B, Table S7‐1). For CRC in the positive HRFQ population (Table S8, Figure S3A) and for advanced neoplasia in the negative FIT population, the same conclusion was observed. And other high‐risk factors for negative FIT advanced neoplasia included male, age over 60 years, urban residence and first‐degree family history of CRC. These populations should be offered timely colonoscopy (Table S7–2).

Although different outcome variables (CRC, advanced‐stage/early‐stage CRC) and subgroup data (FIT positive/negative) could be considered as sensitivity analyses, we further performed sensitivity analyses by setting different reference groups in the time intervals. In sensitivity analyses (Table S11, Figure S4), The finding of increased overall CRC detection rates with time interval >6 months persisted regardless of reference group definition. When 4265 originally‐excluded patients who had a history of colonic polyps prior to FIT screening were included (4216 cases were still excluded due to lack of FIT or other information), the incidence of CRC detection also increased with one time interval >6 months.

## 4 DISCUSSION

In this study, we analyzed the results of 49,810 people at high risk of CRC who underwent colonoscopy in the CRC screening program in Tianjin, China, and no significant increase in CRC risk was found with colonoscopy within 6 months after screening. When colonoscopy was performed more than 6 months later, the detection rate of CRC increased significantly (OR_min_ 1.70, 95% CI 1.34–2.14), regardless of positive FIT or HRFQ results. Furthermore, CRC detection in populations with a positive FIT was significantly increased at longer intervals (OR_min_ 1.89, 95% CI 1.46–2.43). These results provide an important reference for the optimal time of colonoscopy in high‐risk CRC populations.

For a positive FIT/FOBT or positive HRFQ population, a well‐planned colonoscopy can effectively reduce the incidence of CRC and mortality of advanced CRC.^2^, ^3^, ^22^ Although colonoscopy is technically mature with minimal risks, some high‐risk groups still refuse or delay colonoscopy due to multiple factors such as the need for bowel preparation, fear or discomfort.^23^ Our CRC screening program provided free colonoscopy for high‐risk groups, and encouraged their participation by timely reminders by annual follow‐ups. Such strategies improved the participation rate but contributed to a long interval time. In our study, 39.41% and 79.56% of the high‐risk population underwent colonoscopy within 1 month and 6 months, respectively, while the proportion of patients with an interval longer than 6 months was higher than reported in the literature.^13^, ^24^

Current evidence suggests that increased time interval between abnormal FIT/FOBT and colonoscopy leads to increased prevalence of CRC, especially advanced CRC.^24^ Such delay may result in a gradual increase of advanced‐stage cancers, especially with longer intervals.^25^, ^26^ Consistently, a study with more than 200,000 cases noted that prolonged intervals were associated with an increased risk of CRC incidence, death, and advanced CRC.^8^

Currently, the appropriate time interval is subject to significant heterogeneity due to differences in countries, medical institutions, medical personnel.^9^, ^14^, ^27^, ^28^ This is also reflected in different CRC screening guidelines, and the requirements for the interval between colonoscopies after FIT are very broad and diverse. The recommended intervals may vary from 1 month to several months, or no specific interval.^3^, ^29^ Our data found that the incidence of CRC was higher with longer intervals, but the risk within 6 months was not statistically significantly different from that within 1 month. If colonoscopy was conducted more than 6 months later, the incidence of CRC was increased compared with colonoscopy within 6 months (OR_min_ 1.70, 95% CI 1.34–2.14), and the incidence of detection of advanced CRC was increased (OR_min_ 2.28, 95% CI 1.46–3.44). Therefore, we recommend that colonoscopy for high‐risk populations should not exceed 6 months.

Risk stratification of various types of CRC plays a crucial role in screening.^30^ Our study included not only the high‐risk population with a positive FIT, but also those with negative FIT results. It has been suggested that colonoscopy can be delayed for more than 6 months if the FIT is negative in high‐risk populations without a history of advanced adenoma.^15^ In our study, the high‐risk population with negative FIT with an interval >12 months also had an increased incidence of CRC (OR_min_ 1.82, 95% CI 1.10–2.97). But the significance of the risk stratification questionnaire for detecting CRC and advanced adenomas may be inferior to FIT.^31^ Our questionnaire covered many factors of CRC occurrence regarding a history of appendiceal or gallbladder surgery, which was supported by considerable studies.^32^ However, these factors often indicate the long‐term risk of CRC and do not indicate the necessity of colonoscopy in the short term. After all, the occurrence of CRC is a long‐term process, and we cannot predict the occurrence of CRC in high‐risk groups with a negative FIT. Hence, the significance of the questionnaire^33^ and the value of each factor^18^ require further evaluation.

Even in people at low risk of CRC,^34^ FIT is also very sensitive for its detection,^35^ although there may be some variation with different tumors site.^36^, ^37^ The occurrence of CRC is widely acknowledged to involve substantial genetic and molecular mechanism, but most colorectal tumors originate from polyps that originate from normal colonic mucosa, develop into advanced adenomas, and finally into CRC, which is thought to take years or more.^38^ In the process, advanced adenomas represent an important intermediate stage for adenomas to develop into CRC. The long process may be gradually accelerated. Many studies have pointed out that delayed colonoscopy after positive FIT leads to increased detection of CRC, which suggests that a proportion of patients in the FIT‐positive population may soon experience disease progression.^39^ As for the early stages of colorectal carcinogenesis, the time required from polyp formation to advanced adenoma is long, and the detection rates of polyps and advanced adenoma were not significantly changed with a time interval of 1 year or longer.

There are many uncertainties in the development of CRC, and the association between detection rate and interval time is not a simple linear relationship. The main factors affecting CRC detection rate may be the transformation rate from advanced adenoma to CRC and from polyp to advanced adenoma. The annual progress rate from advanced adenoma to cancer is around 5%, and gradually increases with age. Even though FIT is not sensitive enough in such a long interval, it is still possible to recognize advanced adenoma before carcinogenesis. Therefore, we carried out CRC screening on a 3‐year cycle to improve the screening effect of CRC as much as possible.

Compared with colonoscopy within 1 month, the risk of CRC and advanced adenoma detection in high‐risk populations was slightly reduced at intervals of less than 6 months. The possible reason might be the presence of prompting factor of colonoscopy among high‐risk people despite the screening program focused on asymptomatic people, which was consistent with conclusions of other studies.^24^ The advanced adenoma detection rate at an interval of 6–24 months did not increase, while there showed an upward trend after 36 months (OR 1.37, 95% CI 1.18–1.59). This finding also confirms the findings of other studies suggesting that when FIT is abnormal, a non‐advanced adenoma may take 10–12 months and even longer to develop into advanced adenomas and finally to CRC, providing guidance for selecting the CRC screening cycle. As for positive FIT cases with long time intervals but normal colonoscopy results, the positive FIT may not be associated with colorectal neoplastic diseases.^6^, ^8^

There were some limitations in our study. First, our data was based on a large‐scale screening among the population. Although we tried to collect all colonoscopy data, the overall colonoscopy compliance was not high, which might bias the sample data. Moreover, the information and influencing factors of patients in our statistics were limited; in some cases, patients might have undergone colonoscopy due to factors we failed to cover. Moreover, we were not able to obtain all CRC staging data because these data were not in our database for CRC screening. Last, no follow‐up was conducted to assess patient prognosis and hence, we could not assess the impact of delayed colonoscopy on survival, although we provided compelling evidence of the benefit of timely colonoscopy in high‐risk population.^24^

By analyzing CRC screening data in Tianjin, we confirmed that colonoscopy should be performed in time for asymptomatic people at high risk of CRC, especially those with a positive FIT. With an interval of more than 6 months, the risk of CRC detection was increased, while with an interval of more than 36 months, the risk of advanced adenomas and non‐advanced adenoma detection is increased in high‐risk groups. Therefore, it is essential to encourage high‐risk groups to undergo colonoscopy as early as possible using follow‐up or repeat screening.

## AUTHOR CONTRIBUTIONS

**Mingqing Zhang:** Conceptualization (equal); formal analysis (equal); funding acquisition (equal); investigation (equal); methodology (equal); project administration (equal); resources (equal); validation (equal); writing – original draft (lead). **Yongdan Zhang:** Formal analysis (equal); investigation (equal); methodology (equal); writing – review and editing (equal). **Wen Zhang:** Data curation (equal). **Lizhong Zhao:** Formal analysis (equal); investigation (equal); methodology (equal); project administration (equal); writing – review and editing (equal). **Haoren Jing:** Investigation (equal); validation (equal). **Xiaojing Wu:** Formal analysis (equal); validation (equal). **Lu Guo:** Data curation (equal). **Haixiang Zhang:** Data curation (equal); software (equal). **Yong Zhang:** Data curation (equal); software (equal). **Siwei Zhu:** Conceptualization (equal); supervision (equal); visualization (equal). **Shiwu Zhang:** Conceptualization (equal); resources (equal); visualization (equal); writing – review and editing (equal). **Xipeng Zhang:** Conceptualization (equal); funding acquisition (equal); resources (equal); supervision (equal).

## FUNDING INFORMATION

This study was funded by Foundation of Tianjin Union Medical Center (grant numbers: 2016RMNK002, 2019ZDXK04, and 2022GCXK005); This work was funded by the foundation of committee on science and technology of Tianjin (21JCYBJC01090 and 21JCZDJC00990); This work was funded by Tianjin Key Medical Discipline (Specialty) Construction Project (Number: TJYXZDXK‐044A). The funding source had no role in study design, data collection, analysis, or interpretation, report writing, or the decision to submit this paper for publication.

## CONFLICT OF INTEREST STATEMENT

The authors disclose no conflicts.

## ETHICS STATEMENT

The approval of a research ethics committee was not required, since this study is a descriptive analysis of individual data without any direct or indirect intervention on patients. And all investigations and methods used were in accordance with the Declaration of Helsinki.

## Supporting information


**Data S1:** Supporting Information

## Data Availability

The data, analytic methods, and study materials will not available to other researchers
