# Supplementary material for: Postponing colonoscopy for 6 months in high‐risk population increases colorectal cancer detection in China
Source: Cancer Med. 2023 Mar 23;12(10):11816–27. doi: 10.1002/cam4.5850 (PMC10242305; doi:10.1002/cam4.5850)
Supplement: Supplementary file 1 — Data S1: Supporting Information [file CAM4-12-11816-s001.docx]

**Postponing colonoscopy for 6 months in high-risk population increases colorectal cancer detection in China**

High-risk population aged 40–74 years who had FIT and/or HRFQ in Tianjin, n=72,269

Final included cases, n=49,810

Non-CRC, n=48,482

CRC, n=1,328

Exclusions

History of CRC, n=744

Non-FIT, n=12,818

HRFQ, Residential area, Education, Occupation Missing values and unknown, n=368

Other and poor bowel preparation, n=48

History of colonic polyps, n=8,481

Non-AA, n=45332

AA, n=3150

**Supplemental Figure 1. Study population screening flow chart.**

**NOTE:** The CRC screening program was developed for asymptomatic population aged 40–74 in a 3-year cycle since 2012 in Tianjin, China. Based on the screening database, we extracted data of all high-risk individuals undergoing colonoscopy from 2012 to 2020, that is, high-risk population who completed questionnaire, FIT, and colonoscopy. We excluded those with history of CRC or colonic polyps and who did not undertake colonoscopy during the study period due to poor bowel preparation or other (haemostasis).


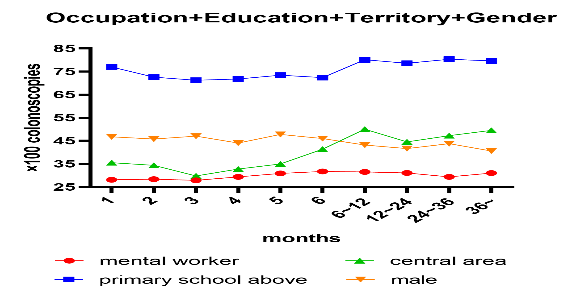

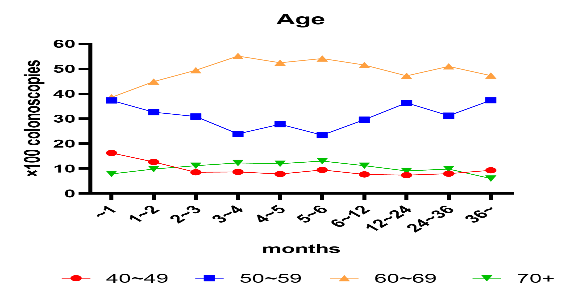

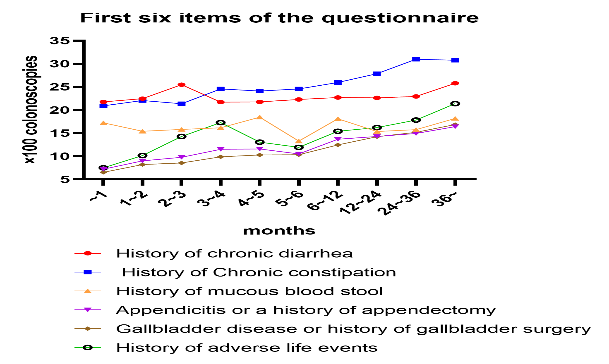

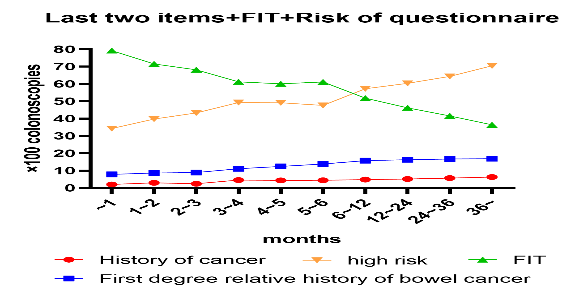


**Supplemental Figure 2.tif Characteristics and CRC outcomes in high-risk population undergoing colonoscopy.**


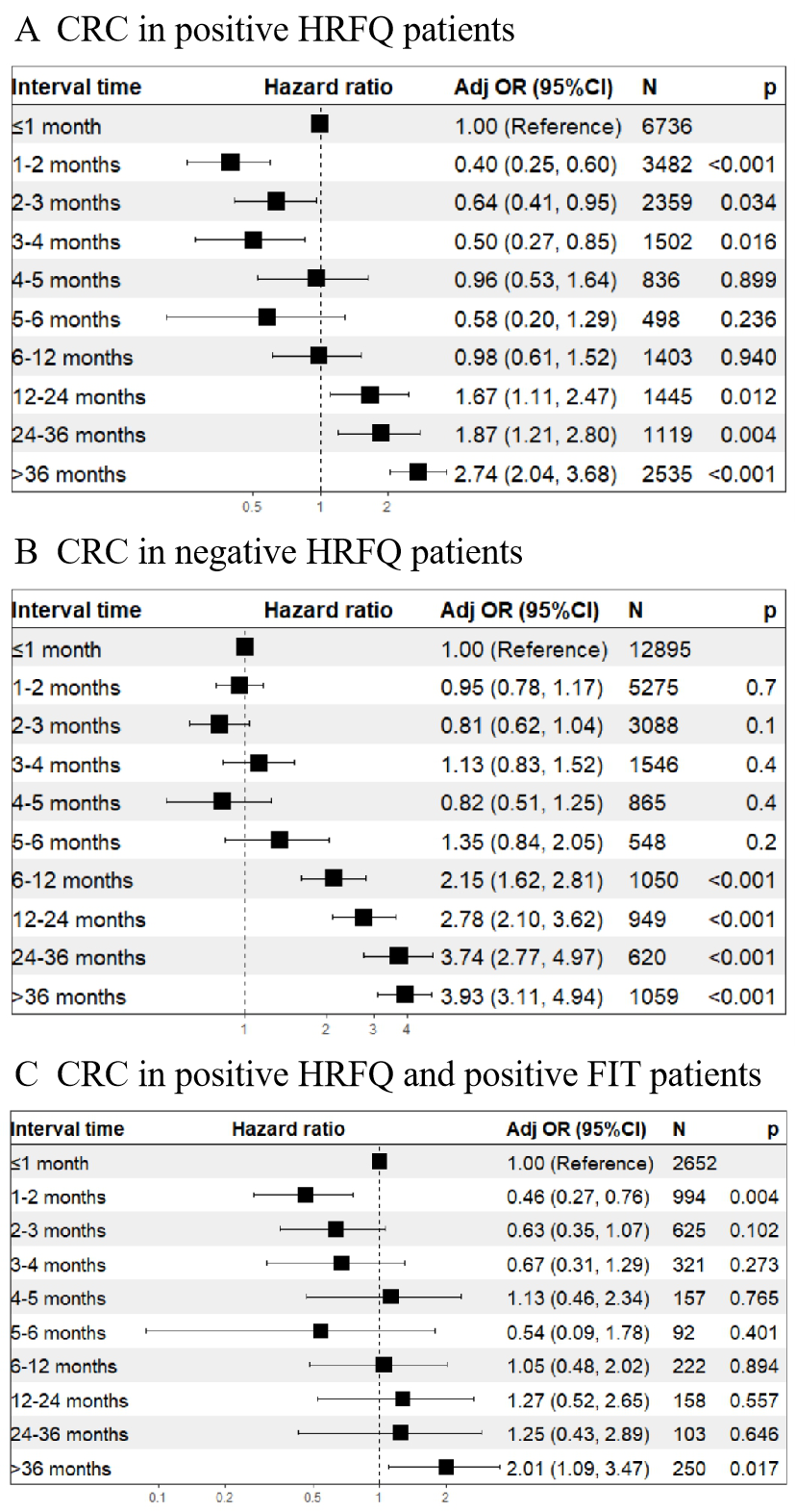


**Supplemental Figure 3.tif Time to colonoscopy after identified as positive HRFQ (A), negative HRFQ (B), positive HRFQ and positive FIT (C) and adjusted incidence of CRC.**


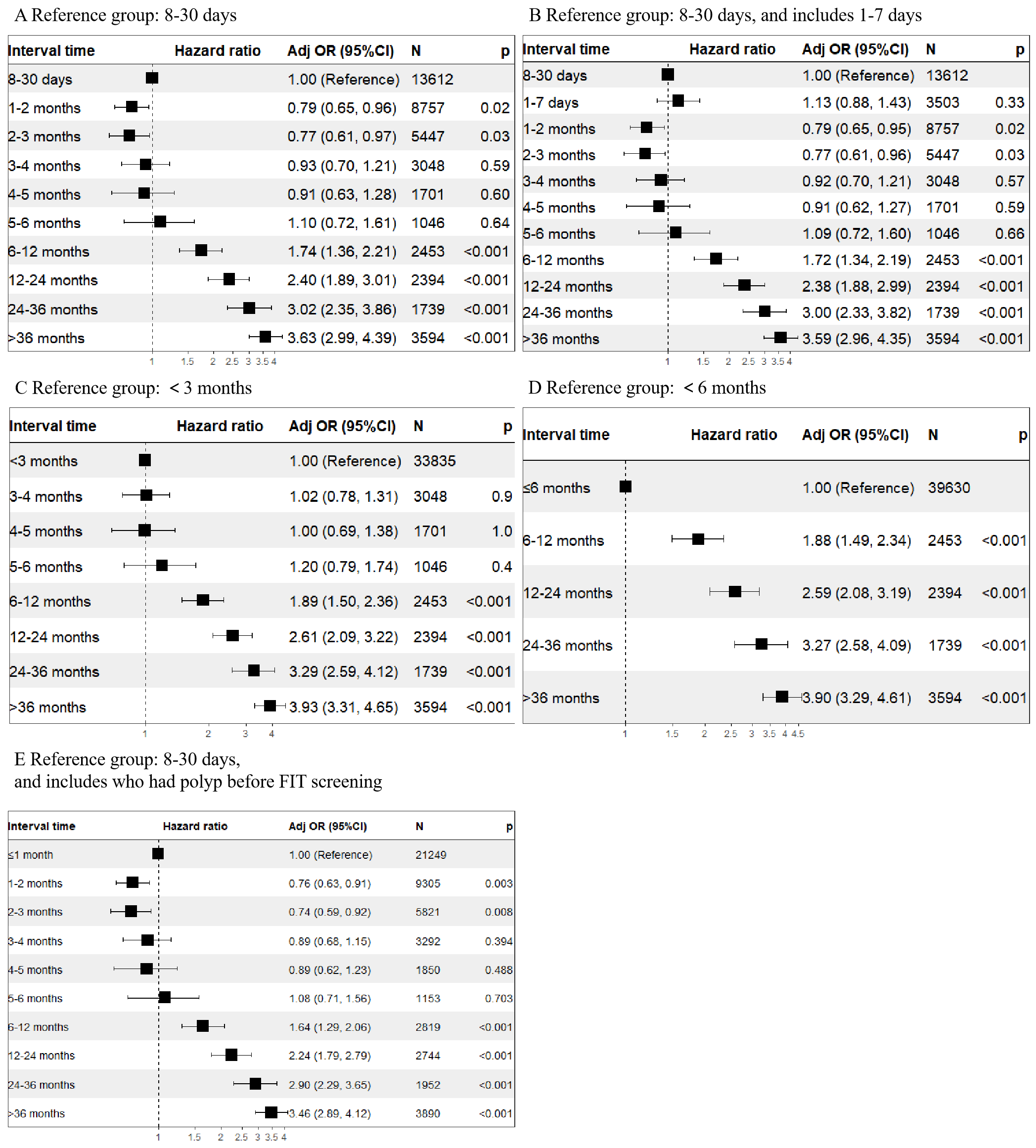


**Supplemental Figure 4.tif Time to colonoscopy after identified as high-risk population and adjusted incidence of CRC: sensitivity analyses. (A) 8-30 days; (B) 8-30 days, and includes 1-7 days; (C) ＜3 months; (D) ＜6 months; (E) ＜1 months, and includes who had polyp before FIT screening.**

**Supplemental Table 1. Time to colonoscopy after identified as high-risk population and adjusted incidence of CRC.**

|  |  | | **Unadjusted** | | | | **Adjusted** | | | |
| --- | --- | --- | --- | --- | --- | --- | --- | --- | --- | --- |
|  | **n** | **%** | **Beta** | **OR** | **95%CI** | **p Value** | **Beta** | **OR** | **95%CI** | **p Value** |
| **Time to Colonoscopy after FIT, mo** |  |  |  |  |  |  |  |  |  |  |
| **<1** | 19631 | 39.41 | Reference |  |  |  |  |  |  |  |
| **1-2** | 8757 | 17.58 | -0.21997 | 0.8 | 0.67-0.96 | 0.0168 | -0.24946 | 0.78 | 0.65-0.93 | 0.00712 |
| **2-3** | 5447 | 10.94 | -0.23011 | 0.79 | 0.64-0.98 | 0.0382 | -0.27252 | 0.76 | 0.61-0.94 | 0.01505 |
| **3-4** | 3048 | 6.12 | -0.08743 | 0.92 | 0.70-1.18 | 0.5142 | -0.0897 | 0.91 | 0.69-1.18 | 0.50812 |
| **4-5** | 1701 | 3.41 | -0.12396 | 0.88 | 0.61-1.23 | 0.4843 | -0.10964 | 0.9 | 0.62-1.25 | 0.53961 |
| **5-6** | 1046 | 2.10 | 0.10813 | 1.11 | 0.73-1.62 | 0.59 | 0.07891 | 1.08 | 0.71-1.58 | 0.69669 |
| **6-12** | 2453 | 4.92 | 0.4594 | 1.58 | 1.25-1.98 | 9.78E-05 | 0.53219 | 1.7 | 1.34-2.14 | 9.73E-06 |
| **12-24** | 2394 | 4.81 | 0.63686 | 1.89 | 1.51-2.34 | 1.06E-08 | 0.85335 | 2.35 | 1.87-2.92 | 8.07E-14 |
| **24-36** | 1739 | 3.49 | 0.83076 | 2.3 | 1.81-2.88 | 2.06E-12 | 1.08442 | 2.96 | 2.32-3.74 | < 2e-16 |
| **>36** | 3594 | 7.22 | 0.89678 | 2.45 | 2.06-2.90 | < 2e-16 | 1.26297 | 3.54 | 2.95-4.23 | < 2e-16 |
| **Gender** |  |  |  |  |  |  |  |  |  |  |
| **Female** | 27155 | 54.52 | Reference |  |  |  |  |  |  |  |
| **Male** | 22655 | 45.48 | 0.43811 | 1.55 | 1.39-1.73 | 5.38E-15 | 0.33593 | 1.4 | 1.25-1.57 | 5.09E-09 |
| **Age** |  |  |  |  |  |  |  |  |  |  |
| **40-59** | 22898 | 45.97 | Reference |  |  |  |  |  |  |  |
| **60-** | 26912 | 54.03 | 1.09582 | 2.99 | 2.63-3.41 | <2e-16 | 0.96802 | 2.63 | 2.31-3.01 | < 2e-16 |
| **Education** |  |  |  |  |  |  |  |  |  |  |
| **Elementary school above** | 37648 | 75.58 | Reference |  |  |  |  |  |  |  |
| **Elementary School/below** | 12162 | 24.42 | -0.08237 | 0.92 | 0.81-1.05 | 0.213 |  |  |  |  |
| **Occupation** |  |  |  |  |  |  |  |  |  |  |
| **mental work** | 14418 | 28.95 | Reference |  |  |  |  |  |  |  |
| **manual work** | 35392 | 71.05 | 0.02041 | 1.02 | 0.91-1.15 | 0.74 |  |  |  |  |
| **Residential area** |  |  |  |  |  |  |  |  |  |  |
| **central urban** | 18524 | 37.19 | Reference |  |  |  |  |  |  |  |
| **agriculture-related areas** | 31286 | 62.81 | -0.58878 | 0.56 | 0.50-0.62 | <2e-16 | -0.5229 | 0.59 | 0.53-0.66 | < 2e-16 |
| **FIT** |  |  |  |  |  |  |  |  |  |  |
| **Negative** | 16341 | 32.81 | Reference |  |  |  |  |  |  |  |
| **Positive** | 33469 | 67.19 | 0.94218 | 2.57 | 2.22-2.98 | <2e-16 | 1.1492 | 3.16 | 2.62-3.81 | < 2e-16 |
| **History of chronic diarrhea** |  |  |  |  |  |  |  |  |  |  |
| **No** | 38490 | 77.27 | Reference |  |  |  |  |  |  |  |
| **Yes** | 11320 | 22.73 | -0.42486 | 0.65 | 0.56-0.76 | 1.56E-08 | -0.09252 | 0.91 | 0.78-1.06 | 0.24118 |
| **History of chronic constipation** |  |  |  |  |  |  |  |  |  |  |
| **No** | 38242 | 76.78 | Reference |  |  |  |  |  |  |  |
| **Yes** | 11568 | 23.22 | -0.48209 | 0.62 | 0.53-0.71 | 2.04E-10 | -0.2518 | 0.78 | 0.66-0.91 | 0.0015 |
| **History of bloody mucous stools** |  |  |  |  |  |  |  |  |  |  |
| **No** | 41530 | 83.38 | Reference |  |  |  |  |  |  |  |
| **Yes** | 8280 | 16.62 | 0.0882 | 1.09 | 0.95-1.26 | 0.225 |  |  |  |  |
| **History of chronic appendicitis or appendectomy** |  |  |  |  |  |  |  |  |  |  |
| **No** | 44890 | 90.12 | Reference |  |  |  |  |  |  |  |
| **Yes** | 4820 | 9.68 | -0.40795 | 0.67 | 0.53-0.82 | 0.000199 | -0.18943 | 0.83 | 0.66-1.03 | 0.0956 |
| **History of chronic cholecystitis or gallstones** |  |  |  |  |  |  |  |  |  |  |
| **No** | 45239 | 90.82 | Reference |  |  |  |  |  |  |  |
| **Yes** | 4571 | 9.18 | -0.22114 | 0.8 | 0.65-0.98 | 0.0355 | -0.01647 | 0.98 | 0.79-1.22 | 0.88213 |
| **Adverse life events** |  |  |  |  |  |  |  |  |  |  |
| **No** | 43951 | 88.24 | Reference |  |  |  |  |  |  |  |
| **Yes** | 5859 | 11.76 | -0.24635 | 0.78 | 0.65-0.94 | 0.00927 | 0.06479 | 1.07 | 0.87-1.29 | 0.51727 |
| **History of cancer** |  |  |  |  |  |  |  |  |  |  |
| **No** | 48189 | 96.75 | Reference |  |  |  |  |  |  |  |
| **Yes** | 1621 | 3.25 | -0.22266 | 0.8 | 0.56-1.11 | 0.199 |  |  |  |  |
| **History of CRC in a first-degree relative** |  |  |  |  |  |  |  |  |  |  |
| **No** | 44669 | 89.68 | Reference |  |  |  |  |  |  |  |
| **Yes** | 5141 | 10.32 | -0.50684 | 0.6 | 0.48-0.75 | 5.89E-06 | -0.16772 | 0.85 | 0.66-1.07 | 0.16758 |

NOTE: The number of participants in this logistic regression was 49810.

**Supplemental Table 2. Time to colonoscopy after identified as high-risk population and adjusted incidence of advanced adenoma.**

|  |  |  | **Unadjusted** | | | | **Adjusted** | | | |
| --- | --- | --- | --- | --- | --- | --- | --- | --- | --- | --- |
|  | **n** | **%** | **Beta** | **OR** | **95%CI** | **p Value** | **Beta** | **OR** | **95%CI** | **p Value** |
| **Time to Colonoscopy after FIT, mo** |  |  |  |  |  |  |  |  |  |  |
| **<1** | 19175 | 39.55 | Reference |  |  |  |  |  |  |  |
| **1-2** | 8593 | 17.72 | -0.09227 | 0.91 | 0.82-1.01 | 0.07928 | -0.11206 | 0.89 | 0.80-0.99 | 0.036524 |
| **2-3** | 5346 | 11.03 | -0.17941 | 0.84 | 0.73-0.95 | 0.00565 | -0.20911 | 0.81 | 0.71-0.92 | 0.001585 |
| **3-4** | 2983 | 6.15 | -0.06742 | 0.93 | 0.80-1.09 | 0.39649 | -0.06821 | 0.93 | 0.79-1.09 | 0.402286 |
| **4-5** | 1666 | 3.44 | -0.22998 | 0.79 | 0.64-0.98 | 0.03731 | -0.24486 | 0.78 | 0.62-0.97 | 0.029221 |
| **5-6** | 1019 | 2.10 | -0.10467 | 0.9 | 0.69-1.16 | 0.42857 | -0.14 | 0.87 | 0.66-1.12 | 0.297559 |
| **6-12** | 2364 | 4.88 | -0.26225 | 0.77 | 0.64-0.92 | 0.00589 | -0.22901 | 0.8 | 0.65-0.96 | 0.018788 |
| **12-24** | 2291 | 4.73 | -0.17257 | 0.84 | 0.70-1.01 | 0.06398 | -0.02433 | 0.98 | 0.81-1.17 | 0.799462 |
| **24-36** | 1649 | 3.40 | -0.10023 | 0.9 | 0.73-1.11 | 0.34083 | 0.06238 | 1.06 | 0.86-1.31 | 0.564488 |
| **>36** | 3396 | 7.00 | 0.04394 | 1.04 | 0.91-1.20 | 0.54257 | 0.31447 | 1.37 | 1.18-1.59 | 3.69E-05 |
| **Gender** |  |  |  |  |  |  |  |  |  |  |
| **Female** | 26572 | 54.81 | Reference |  |  |  |  |  |  |  |
| **Male** | 21910 | 45.19 | 0.79049 | 2.2 | 2.05-2.38 | <2e-16 | 0.70542 | 2.02 | 1.87-2.19 | < 2e-16 |
| **Age** |  |  |  |  |  |  |  |  |  |  |
| **40-59** | 22598 | 46.61 | Reference |  |  |  |  |  |  |  |
| **60-** | 25884 | 53.39 | 0.83411 | 2.3 | 2.13-2.49 | <2e-16 | 0.75931 | 2.14 | 1.97-2.32 | < 2e-16 |
| **Education** |  |  |  |  |  |  |  |  |  |  |
| **Elementary school above** | 36625 | 75.54 | Reference |  |  |  |  |  |  |  |
| **Elementary School/below** | 11857 | 24.46 | -0.2172 | 0.8 | 0.74-0.88 | 1.51E-06 | -0.15174 | 0.86 | 0.78-0.95 | 0.003075 |
| **Occupation** |  |  |  |  |  |  |  |  |  |  |
| **mental work** | 14039 | 28.96 | Reference |  |  |  |  |  |  |  |
| **manual work** | 34443 | 71.04 | -0.02767 | 0.97 | 0.90-1.05 | 0.494 |  |  |  |  |
| **Residential area** |  |  |  |  |  |  |  |  |  |  |
| **central urban** | 17844 | 36.81 | Reference |  |  |  |  |  |  |  |
| **agriculture-related areas** | 30638 | 63.19 | -0.42976 | 0.65 | 0.61-0.70 | <2e-16 | -0.38347 | 0.68 | 0.63-0.74 | < 2e-16 |
| **FIT** |  |  |  |  |  |  |  |  |  |  |
| **Negative** | 16125 | 33.26 | Reference |  |  |  |  |  |  |  |
| **Positive** | 32357 | 66.74 | 0.81851 | 2.27 | 2.07-2.49 | <2e-16 | 0.7054 | 2.02 | 1.81-2.27 | < 2e-16 |
| **History of chronic diarrhea** |  |  |  |  |  |  |  |  |  |  |
| **No** | 37378 | 77.10 | Reference |  |  |  |  |  |  |  |
| **Yes** | 11104 | 22.90 | -0.40804 | 0.66 | 0.60-0.73 | <2e-16 | -0.19307 | 0.82 | 0.74-0.91 | 0.000278 |
| **History of chronic constipation** |  |  |  |  |  |  |  |  |  |  |
| **No** | 37125 | 76.57 | Reference |  |  |  |  |  |  |  |
| **Yes** | 11357 | 23.43 | -0.48995 | 0.61 | 0.56-0.67 | <2e-16 | -0.23137 | 0.79 | 0.72-0.88 | 9.80E-06 |
| **History of bloody mucous stools** |  |  |  |  |  |  |  |  |  |  |
| **No** | 40439 | 83.41 | Reference |  |  |  |  |  |  |  |
| **Yes** | 8043 | 16.59 | -0.35361 | 0.7 | 0.63-0.78 | 1.68E-10 | -0.05612 | 0.95 | 0.84-1.06 | 0.350273 |
| **History of chronic appendicitis or appendectomy** |  |  |  |  |  |  |  |  |  |  |
| **No** | 43653 | 90.04 | Reference |  |  |  |  |  |  |  |
| **Yes** | 4829 | 9.96 | -0.2881 | 0.75 | 0.65-0.86 | 2.48E-05 | -0.0482 | 0.95 | 0.83-1.09 | 0.499486 |
| **History of chronic cholecystitis or gallstones** |  |  |  |  |  |  |  |  |  |  |
| **No** | 44011 | 90.78 | Reference |  |  |  |  |  |  |  |
| **Yes** | 4471 | 9.22 | -0.16105 | 0.85 | 0.74-0.97 | 0.0171 | 0.12832 | 1.14 | 0.99-1.31 | 0.073155 |
| **Adverse life events** |  |  |  |  |  |  |  |  |  |  |
| **No** | 42749 | 88.17 | Reference |  |  |  |  |  |  |  |
| **Yes** | 5733 | 11.83 | -0.44948 | 0.64 | 0.56-0.73 | 1.96E-11 | -0.14487 | 0.87 | 0.75-0.99 | 0.03934 |
| **History of cancer** |  |  |  |  |  |  |  |  |  |  |
| **No** | 46896 | 96.73 | Reference |  |  |  |  |  |  |  |
| **Yes** | 1586 | 3.27 | -0.37528 | 0.69 | 0.54-0.86 | 0.00197 | -0.03003 | 0.97 | 0.75-1.23 | 0.812538 |
| **History of CRC in a first-degree relative** |  |  |  |  |  |  |  |  |  |  |
| **No** | 43428 | 89.58 | Reference |  |  |  |  |  |  |  |
| **Yes** | 5054 | 10.42 | -0.04195 | 0.96 | 0.85-1.08 | 0.493 |  |  |  |  |

NOTE: The number of participants in this logistic regression was 48482.

**Supplemental Table 3. Time to colonoscopy after identified as high-risk population and adjusted incidence of non-advanced adenoma.**

|  |  |  | **Unadjusted** | | | | **Adjusted** | | | |
| --- | --- | --- | --- | --- | --- | --- | --- | --- | --- | --- |
|  | **n** | **%** | **Beta** | **OR** | **95%CI** | **p Value** | **Beta** | **OR** | **95%CI** | **p Value** |
| **Time to Colonoscopy after FIT, mo** |  |  |  |  |  |  |  |  |  |  |
| **<1** | 17847 | 39.37 | Reference |  |  |  |  |  |  |  |
| **1-2** | 8047 | 17.75 | -0.05316 | 0.95 | 0.90-1.00 | 0.05468 | -0.06712 | 0.94 | 0.88-0.99 | 0.018046 |
| **2-3** | 5033 | 11.10 | -0.15269 | 0.86 | 0.80-0.92 | 4.40E-06 | -0.19065 | 0.83 | 0.77-0.88 | 2.63E-08 |
| **3-4** | 2789 | 6.15 | -0.04009 | 0.96 | 0.88-1.04 | 0.33919 | -0.07737 | 0.93 | 0.85-1.01 | 0.074063 |
| **4-5** | 1573 | 3.47 | -0.15845 | 0.85 | 0.77-0.95 | 0.00396 | -0.2178 | 0.8 | 0.72-0.90 | 0.000112 |
| **5-6** | 955 | 2.11 | -0.02647 | 0.97 | 0.85-1.11 | 0.6985 | -0.09442 | 0.91 | 0.79-1.04 | 0.177264 |
| **6-12** | 2236 | 4.93 | -0.141 | 0.87 | 0.79-0.95 | 0.00257 | -0.17168 | 0.84 | 0.77-0.93 | 0.000386 |
| **12-24** | 2156 | 4.76 | -0.10386 | 0.9 | 0.82-0.99 | 0.02818 | -0.07252 | 0.93 | 0.84-1.02 | 0.139135 |
| **24-36** | 1545 | 3.41 | 0.12593 | 1.13 | 1.02-1.26 | 0.01926 | 0.138 | 1.15 | 1.03-1.28 | 0.013382 |
| **>36** | 3151 | 6.95 | 0.17976 | 1.2 | 1.11-1.29 | 4.29E-06 | 0.27088 | 1.31 | 1.21-1.42 | 5.68E-11 |
| **Gender** |  |  |  |  |  |  |  |  |  |  |
| **Female** | 25417 | 56.07 | Reference |  |  |  |  |  |  |  |
| **Male** | 19915 | 43.93 | 0.63094 | 1.88 | 1.81-1.95 | <2e-16 | 0.60958 | 1.84 | 1.77-1.91 | < 2e-16 |
| **Age** |  |  |  |  |  |  |  |  |  |  |
| **40-59** | 21700 | 47.87 | Reference |  |  |  |  |  |  |  |
| **60-** | 23632 | 52.13 | 0.44554 | 1.56 | 1.50-1.62 | <2e-16 | 0.4355 | 1.55 | 1.48-1.61 | < 2e-16 |
| **Education** |  |  |  |  |  |  |  |  |  |  |
| **Elementary school above** | 34133 | 75.30 | Reference |  |  |  |  |  |  |  |
| **Elementary School/below** | 11199 | 24.70 | -0.10071 | 0.9 | 0.87-0.95 | 8.08E-06 | -0.0968 | 0.91 | 0.86-0.95 | 0.000157 |
| **Occupation** |  |  |  |  |  |  |  |  |  |  |
| **mental work** | 13110 | 28.92 | Reference |  |  |  |  |  |  |  |
| **manual work** | 32222 | 71.08 | 0.01606 | 1.02 | 0.97-1.06 | 0.452 |  |  |  |  |
| **Residential area** |  |  |  |  |  |  |  |  |  |  |
| **central urban** | 16379 | 36.13 | Reference |  |  |  |  |  |  |  |
| **agriculture-related areas** | 28953 | 63.87 | -0.15061 | 0.86 | 0.83-0.89 | 5.79E-14 | -0.09542 | 0.91 | 0.87-0.95 | 3.15E-05 |
| **FIT** |  |  |  |  |  |  |  |  |  |  |
| **Negative** | 15536 | 34.27 | Reference |  |  |  |  |  |  |  |
| **Positive** | 29796 | 65.73 | 0.22802 | 1.26 | 1.21-1.31 | <2e-16 | 0.19195 | 1.21 | 1.15-1.28 | 6.09E-12 |
| **History of chronic diarrhea** |  |  |  |  |  |  |  |  |  |  |
| **No** | 34758 | 76.67 | Reference |  |  |  |  |  |  |  |
| **Yes** | 10574 | 23.33 | -0.19591 | 0.82 | 0.79-0.86 | <2e-16 | -0.14975 | 0.86 | 0.82-0.91 | 9.12E-09 |
| **History of chronic constipation** |  |  |  |  |  |  |  |  |  |  |
| **No** | 34484 | 76.07 | Reference |  |  |  |  |  |  |  |
| **Yes** | 10848 | 23.93 | -0.21383 | 0.81 | 0.77-0.84 | <2e-16 | -0.08019 | 0.92 | 0.88-0.97 | 0.001385 |
| **History of bloody mucous stools** |  |  |  |  |  |  |  |  |  |  |
| **No** | 37682 | 83.12 | Reference |  |  |  |  |  |  |  |
| **Yes** | 7650 | 16.88 | -0.16696 | 0.85 | 0.80-0.89 | 1.88E-10 | -0.0421 | 0.96 | 0.91-1.01 | 0.146733 |
| **History of chronic appendicitis or appendectomy** |  |  |  |  |  |  |  |  |  |  |
| **No** | 40748 | 89.89 | Reference |  |  |  |  |  |  |  |
| **Yes** | 4584 | 10.11 | -0.0445 | 0.96 | 0.90-1.02 | 0.167 |  |  |  |  |
| **History of chronic cholecystitis or gallstones** |  |  |  |  |  |  |  |  |  |  |
| **No** | 41114 | 90.70 | Reference |  |  |  |  |  |  |  |
| **Yes** | 4218 | 9.30 | -0.06291 | 0.94 | 0.88-1.00 | 0.0604 |  |  |  |  |
| **Adverse life events** |  |  |  |  |  |  |  |  |  |  |
| **No** | 39853 | 87.91 | Reference |  |  |  |  |  |  |  |
| **Yes** | 5479 | 12.09 | -0.12158 | 0.89 | 0.83-0.94 | 5.13E-05 | -0.02849 | 0.97 | 0.91-1.04 | 0.376803 |
| **History of cancer** |  |  |  |  |  |  |  |  |  |  |
| **No** | 43819 | 96.66 | Reference |  |  |  |  |  |  |  |
| **Yes** | 1513 | 3.34 | -0.206038 | 0.81 | 0.73-0.91 | 0.000196 | -0.0664 | 0.94 | 0.83-1.05 | 0.260062 |
| **History of CRC in a first-degree relative** |  |  |  |  |  |  |  |  |  |  |
| **No** | 40595 | 89.55 | Reference |  |  |  |  |  |  |  |
| **Yes** | 4737 | 10.45 | 0.14264 | 1.15 | 1.08-1.23 | 4.98E-06 | 0.22418 | 1.25 | 1.17-1.34 | 4.05E-10 |

NOTE: The number of participants in this logistic regression was 45332.

**Supplemental Table 4. Time to colonoscopy after identified as high-risk population and adjusted incidence of Advanced-stage CRC.**

|  |  | | **Unadjusted** | | | | **Adjusted** | | | |
| --- | --- | --- | --- | --- | --- | --- | --- | --- | --- | --- |
|  | **n** | **%** | **Beta** | **OR** | **95%CI** | **p Value** | **Beta** | **OR** | **95%CI** | **p Value** |
| **Time to Colonoscopy after FIT, mo** |  |  |  |  |  |  |  |  |  |  |
| **<1** | 19506 | 39.43 | Reference |  |  |  |  |  |  |  |
| **1-2** | 8707 | 17.60 | -0.1885 | 0.83 | 0.56-1.20 | 0.328267 | -0.23216 | 0.79 | 0.54-1.15 | 0.23043 |
| **2-3** | 5417 | 10.95 | -0.2807 | 0.76 | 0.46-1.18 | 0.243171 | -0.33143 | 0.72 | 0.44-1.13 | 0.17018 |
| **3-4** | 3031 | 6.13 | 0.0294 | 1.03 | 0.58-1.70 | 0.913278 | 0.02093 | 1.02 | 0.58-1.69 | 0.93857 |
| **4-5** | 1685 | 3.41 | -0.5489 | 0.58 | 0.20-1.28 | 0.231654 | -0.53611 | 0.59 | 0.21-1.30 | 0.24386 |
| **5-6** | 1038 | 2.10 | 0.4103 | 1.51 | 0.67-2.91 | 0.265933 | 0.36427 | 1.44 | 0.64-2.79 | 0.32559 |
| **6-12** | 2429 | 4.91 | 0.8167 | 2.26 | 1.46-3.40 | 0.000144 | 0.82257 | 2.28 | 1.46-3.44 | 0.00016 |
| **12-24** | 2382 | 4.81 | 1.3406 | 3.82 | 2.67-5.40 | 8.09E-14 | 1.50918 | 4.52 | 3.13-6.44 | < 2e-16 |
| **24-36** | 1719 | 3.47 | 1.0108 | 2.75 | 1.72-4.22 | 9.89E-06 | 1.21305 | 3.36 | 2.08-5.22 | 2.02E-07 |
| **>36** | 3558 | 7.19 | 1.2996 | 3.67 | 2.67-5.00 | 3.95E-16 | 1.58815 | 4.89 | 3.52-6.77 | < 2e-16 |
| **Gender** |  |  |  |  |  |  |  |  |  |  |
| **Female** | 27002 | 54.58 | Reference |  |  |  |  |  |  |  |
| **Male** | 22470 | 45.42 | 0.42142 | 1.52 | 1.23-1.88 | 9.35E-05 | 0.32898 | 1.39 | 1.12-1.73 | 0.00289 |
| **Age** |  |  |  |  |  |  |  |  |  |  |
| **40-59** | 22800 | 46.09 | Reference |  |  |  |  |  |  |  |
| **60-** | 26672 | 53.91 | 1.2405 | 3.46 | 2.68-4.53 | <2e-16 | 1.04513 | 2.84 | 2.18-3.76 | 4.16E-14 |
| **Education** |  |  |  |  |  |  |  |  |  |  |
| **Elementary school above** | 37421 | 75.64 | Reference |  |  |  |  |  |  |  |
| **Elementary School/below** | 12051 | 24.36 | -0.36989 | 0.69 | 0.52-0.90 | 0.00763 | -0.03737 | 0.96 | 0.70-1.31 | 0.81613 |
| **Occupation** |  |  |  |  |  |  |  |  |  |  |
| **mental work** | 14330 | 28.97 | Reference |  |  |  |  |  |  |  |
| **manual work** | 35142 | 71.03 | -0.12686 | 0.88 | 0.70-1.11 | 0.271 |  |  |  |  |
| **Residential area** |  |  |  |  |  |  |  |  |  |  |
| **central urban** | 18419 | 37.23 | Reference |  |  |  |  |  |  |  |
| **agriculture-related areas** | 31053 | 62.77 | -1.02407 | 0.36 | 0.29-0.45 | <2e-16 | -0.90154 | 0.41 | 0.31-0.52 | 2.68E-12 |
| **FIT** |  |  |  |  |  |  |  |  |  |  |
| **Negative** | 16288 | 32.92 | Reference |  |  |  |  |  |  |  |
| **Positive** | 33184 | 67.08 | 0.7187 | 2.05 | 1.58-2.70 | 1.11E-07 | 0.97684 | 2.66 | 1.93-3.69 | 2.93E-09 |
| **History of chronic diarrhea** |  |  |  |  |  |  |  |  |  |  |
| **No** | 38216 | 77.25 | Reference |  |  |  |  |  |  |  |
| **Yes** | 11256 | 22.75 | -0.31901 | 0.73 | 0.55-0.95 | 0.0231 | -0.02544 | 0.97 | 0.72-1.29 | 0.86388 |
| **History of chronic constipation** |  |  |  |  |  |  |  |  |  |  |
| **No** | 37959 | 76.73 | Reference |  |  |  |  |  |  |  |
| **Yes** | 11513 | 23.27 | -0.55955 | 0.57 | 0.42-0.76 | 0.000203 | -0.41019 | 0.66 | 0.48-0.90 | 0.00916 |
| **History of bloody mucous stools** |  |  |  |  |  |  |  |  |  |  |
| **No** | 41245 | 83.37 | Reference |  |  |  |  |  |  |  |
| **Yes** | 8227 | 16.63 | 0.15039 | 1.16 | 0.88-1.51 | 0.273 |  |  |  |  |
| **History of chronic appendicitis or appendectomy** |  |  |  |  |  |  |  |  |  |  |
| **No** | 44577 | 90.11 | Reference |  |  |  |  |  |  |  |
| **Yes** | 4895 | 9.89 | -0.49891 | 0.61 | 0.38-0.91 | 0.0238 | -0.3959 | 0.67 | 0.42-1.03 | 0.08151 |
| **History of chronic cholecystitis or gallstones** |  |  |  |  |  |  |  |  |  |  |
| **No** | 44920 | 90.80 | Reference |  |  |  |  |  |  |  |
| **Yes** | 4552 | 9.20 | 0.12117 | 1.13 | 0.79-1.57 | 0.493 |  |  |  |  |
| **Adverse life events** |  |  |  |  |  |  |  |  |  |  |
| **No** | 43642 | 88.22 | Reference |  |  |  |  |  |  |  |
| **Yes** | 5830 | 11.78 | -0.09627 | 0.91 | 0.64-1.26 | 0.576 |  |  |  |  |
| **History of cancer** |  |  |  |  |  |  |  |  |  |  |
| **No** | 47863 | 96.75 | Reference |  |  |  |  |  |  |  |
| **Yes** | 1609 | 3.25 | 0.05201 | 1.05 | 0.56-1.79 | 0.86 |  |  |  |  |
| **History of CRC in a first-degree relative** |  |  |  |  |  |  |  |  |  |  |
| **No** | 44354 | 89.65 | Reference |  |  |  |  |  |  |  |
| **Yes** | 5118 | 10.35 | -0.54871 | 0.58 | 0.36-0.87 | 0.0129 | -0.42105 | 0.66 | 0.40-1.02 | 0.07692 |

NOTE: The number of participants in this logistic regression was 49472.

**Supplemental Table 5. Time to colonoscopy after identified as high-risk population and adjusted incidence of early-stage CRC.**

|  |  | | **Unadjusted** | | | | **Adjusted** | | | |
| --- | --- | --- | --- | --- | --- | --- | --- | --- | --- | --- |
|  | **n** | **%** | **Beta** | **OR** | **95%CI** | **p Value** | **Beta** | **OR** | **95%CI** | **p Value** |
| **Time to Colonoscopy after FIT, mo** |  |  |  |  |  |  |  |  |  |  |
| **<1** | 19406 | 39.51 | Reference |  |  |  |  |  |  |  |
| **1-2** | 8670 | 17.65 | -0.29595 | 0.74 | 0.57-0.96 | 0.02521 | -0.33349 | 0.72 | 0.55-0.93 | 0.0122 |
| **2-3** | 5396 | 10.99 | -0.25314 | 0.78 | 0.56-1.05 | 0.10631 | -0.30337 | 0.74 | 0.54-1.00 | 0.0547 |
| **3-4** | 3015 | 6.14 | -0.116 | 0.89 | 0.60-1.27 | 0.54075 | -0.12237 | 0.88 | 0.60-1.27 | 0.5225 |
| **4-5** | 1680 | 3.42 | -0.36018 | 0.7 | 0.39-1.15 | 0.19256 | -0.35135 | 0.7 | 0.39-1.17 | 0.206 |
| **5-6** | 1030 | 2.10 | -0.10974 | 0.9 | 0.46-1.57 | 0.72358 | -0.15002 | 0.86 | 0.44-1.51 | 0.6306 |
| **6-12** | 2401 | 4.89 | 0.26175 | 1.3 | 0.90-1.82 | 0.14234 | 0.31158 | 1.37 | 0.94-1.92 | 0.0851 |
| **12-24** | 2336 | 4.76 | 0.48886 | 1.63 | 1.17-2.23 | 0.00295 | 0.70328 | 2.02 | 1.44-2.78 | 2.72E-05 |
| **24-36** | 1695 | 3.45 | 0.83966 | 2.32 | 1.66-3.16 | 2.81E-07 | 1.09785 | 3 | 2.13-4.13 | 6.49E-11 |
| **>36** | 3492 | 7.11 | 0.85294 | 2.35 | 1.84-2.97 | 3.84E-12 | 1.22204 | 3.39 | 2.63-4.36 | < 2e-16 |
| **Gender** |  |  |  |  |  |  |  |  |  |  |
| **Female** | 26847 | 54.65 | Reference |  |  |  |  |  |  |  |
| **Male** | 22274 | 45.35 | 0.4733 | 1.61 | 1.37-1.88 | 3.97E-09 | 0.36807 | 1.44 | 1.23-1.70 | 8.19E-06 |
| **Age** |  |  |  |  |  |  |  |  |  |  |
| **40-59** | 22730 | 46.27 | Reference |  |  |  |  |  |  |  |
| **60-** | 26391 | 53.73 | 1.20995 | 3.35 | 2.78-4.08 | <2e-16 | 1.06847 | 2.91 | 2.39-3.57 | < 2e-16 |
| **Education** |  |  |  |  |  |  |  |  |  |  |
| **Elementary school above** | 37134 | 75.60 | Reference |  |  |  |  |  |  |  |
| **Elementary School/below** | 11987 | 24.40 | -0.2371 | 0.79 | 0.65-0.95 | 0.0164 | -0.0478 | 0.95 | 0.76-1.19 | 0.6761 |
| **Occupation** |  |  |  |  |  |  |  |  |  |  |
| **mental work** | 14219 | 28.95 | Reference |  |  |  |  |  |  |  |
| **manual work** | 34902 | 71.05 | 0.03863 | 1.04 | 0.88-1.24 | 0.663 |  |  |  |  |
| **Residential area** |  |  |  |  |  |  |  |  |  |  |
| **central urban** | 18201 | 37.05 | Reference |  |  |  |  |  |  |  |
| **agriculture-related areas** | 30920 | 62.95 | -0.7764 | 0.46 | 0.39-0.54 | <2e-16 | -0.6975 | 0.5 | 0.41-0.60 | 8.30E-14 |
| **FIT** |  |  |  |  |  |  |  |  |  |  |
| **Negative** | 16220 | 33.02 | Reference |  |  |  |  |  |  |  |
| **Positive** | 32901 | 66.98 | 1.0486 | 2.85 | 2.31-3.57 | <2e-16 | 1.22119 | 3.39 | 2.58-4.48 | < 2e-16 |
| **History of chronic diarrhea** |  |  |  |  |  |  |  |  |  |  |
| **No** | 37927 | 77.21 | Reference |  |  |  |  |  |  |  |
| **Yes** | 11194 | 22.79 | -0.59451 | 0.55 | 0.44-0.69 | 1.94E-07 | -0.25432 | 0.78 | 0.61-0.97 | 0.0328 |
| **History of chronic constipation** |  |  |  |  |  |  |  |  |  |  |
| **No** | 37660 | 76.67 | Reference |  |  |  |  |  |  |  |
| **Yes** | 11461 | 23.33 | -0.45342 | 0.64 | 0.51-0.78 | 2.55E-05 | -0.21694 | 0.8 | 0.64-1.00 | 0.0544 |
| **History of bloody mucous stools** |  |  |  |  |  |  |  |  |  |  |
| **No** | 40960 | 83.39 | Reference |  |  |  |  |  |  |  |
| **Yes** | 8161 | 16.61 | 0.12993 | 1.14 | 0.93-1.39 | 0.206 |  |  |  |  |
| **History of chronic appendicitis or appendectomy** |  |  |  |  |  |  |  |  |  |  |
| **No** | 44248 | 90.08 | Reference |  |  |  |  |  |  |  |
| **Yes** | 4873 | 9.92 | -0.40274 | 0.67 | 0.48-0.90 | 0.0103 | -0.16115 | 0.85 | 0.61-1.16 | 0.3206 |
| **History of chronic cholecystitis or gallstones** |  |  |  |  |  |  |  |  |  |  |
| **No** | 44605 | 90.81 | Reference |  |  |  |  |  |  |  |
| **Yes** | 4516 | 9.19 | -0.29339 | 0.75 | 0.54-1.00 | 0.059 |  |  |  |  |
| **Adverse life events** |  |  |  |  |  |  |  |  |  |  |
| **No** | 43329 | 88.21 | Reference |  |  |  |  |  |  |  |
| **Yes** | 5792 | 11.79 | -0.2764 | 0.76 | 0.57-0.98 | 0.0442 | 0.07577 | 1.08 | 0.80-1.42 | 0.6006 |
| **History of cancer** |  |  |  |  |  |  |  |  |  |  |
| **No** | 47524 | 96.75 | Reference |  |  |  |  |  |  |  |
| **Yes** | 1597 | 3.25 | -0.65793 | 0.52 | 0.27-0.89 | 0.0311 | -0.36408 | 0.69 | 0.35-1.23 | 0.2474 |
| **History of CRC in a first-degree relative** |  |  |  |  |  |  |  |  |  |  |
| **No** | 44025 | 89.63 | Reference |  |  |  |  |  |  |  |
| **Yes** | 5096 | 10.37 | -0.50332 | 0.6 | 0.43-0.82 | 0.00169 | -0.15579 | 0.86 | 0.60-1.19 | 0.3718 |

NOTE: The number of participants in this logistic regression was 49121.

**Supplemental Table 6. Time to colonoscopy after a positive FIT and the adjusted incidence of CRC.**

|  |  | | **Unadjusted** | | | | **Adjusted** | | | |
| --- | --- | --- | --- | --- | --- | --- | --- | --- | --- | --- |
|  | **n** | **%** | **Beta** | **OR** | **95%CI** | **p Value** | **Beta** | **OR** | **95%CI** | **p Value** |
| **Time to Colonoscopy after FIT, mo** |  |  |  |  |  |  |  |  |  |  |
| **<1** | 15547 | 46.45 | Reference |  |  |  |  |  |  |  |
| **1-2** | 6269 | 18.73 | -0.06955 | 0.93 | 0.77-1.12 | 0.4649 | -0.1603 | 0.85 | 0.70-1.03 | 0.09489 |
| **2-3** | 3713 | 11.09 | -0.10793 | 0.9 | 0.71-1.13 | 0.3616 | -0.25343 | 0.78 | 0.61-0.98 | 0.03371 |
| **3-4** | 1867 | 5.58 | 0.19369 | 1.21 | 0.91-1.58 | 0.1676 | 0.02992 | 1.03 | 0.77-1.35 | 0.83273 |
| **4-5** | 1022 | 3.05 | 0.02923 | 1.03 | 0.68-1.49 | 0.8826 | -0.13992 | 0.87 | 0.58-1.26 | 0.48255 |
| **5-6** | 640 | 1.91 | 0.35357 | 1.42 | 0.91-2.12 | 0.0984 | 0.17444 | 1.19 | 0.76-1.78 | 0.41824 |
| **6-12** | 1272 | 3.80 | 0.82868 | 2.29 | 1.77-2.93 | 1.34E-10 | 0.63864 | 1.89 | 1.46-2.43 | 1.03E-06 |
| **12-24** | 1107 | 3.31 | 1.00526 | 2.73 | 2.11-3.49 | 4.49E-15 | 0.91268 | 2.49 | 1.92-3.20 | 2.29E-12 |
| **24-36** | 723 | 2.16 | 1.28395 | 3.61 | 2.73-4.71 | < 2e-16 | 1.16822 | 3.22 | 2.42-4.21 | < 2e-16 |
| **>36** | 1309 | 3.91 | 1.34155 | 3.82 | 3.09-4.70 | < 2e-16 | 1.25309 | 3.5 | 2.82-4.32 | < 2e-16 |
| **Gender** |  |  |  |  |  |  |  |  |  |  |
| **Female** | 17259 | 51.57 | Reference |  |  |  |  |  |  |  |
| **Male** | 16210 | 48.43 | 0.34937 | 1.42 | 1.26-1.60 | 1.38E-08 | 0.27055 | 1.31 | 1.16-1.48 | 1.90E-05 |
| **Age** |  |  |  |  |  |  |  |  |  |  |
| **40-59** | 14976 | 44.75 | Reference |  |  |  |  |  |  |  |
| **60-** | 18493 | 55.25 | 1.11135 | 3.04 | 2.63-3.52 | <2e-16 | 1.02664 | 2.79 | 2.40-3.25 | < 2e-16 |
| **Education** |  |  |  |  |  |  |  |  |  |  |
| **Elementary school above** | 24650 | 73.65 | Reference |  |  |  |  |  |  |  |
| **Elementary School/below** | 8819 | 26.35 | -0.18511 | 0.83 | 0.72-0.96 | 0.0105 | -0.03389 | 0.97 | 0.82-1.14 | 0.6868 |
| **Occupation** |  |  |  |  |  |  |  |  |  |  |
| **mental work** | 9163 | 27.38 | Reference |  |  |  |  |  |  |  |
| **manual work** | 24306 | 72.62 | -0.05349 | 0.95 | 0.83-1.08 | 0.429 |  |  |  |  |
| **Residential area** |  |  |  |  |  |  |  |  |  |  |
| **central urban** | 11354 | 33.92 | Reference |  |  |  |  |  |  |  |
| **agriculture-related areas** | 22115 | 66.08 | -0.69517 | 0.5 | 0.44-0.56 | <2e-16 | -0.51165 | 0.6 | 0.52-0.69 | 6.88E-13 |
| **History of chronic diarrhea** |  |  |  |  |  |  |  |  |  |  |
| **No** | 28573 | 85.37 | Reference |  |  |  |  |  |  |  |
| **Yes** | 4896 | 14.63 | -0.18771 | 0.83 | 0.69-0.99 | 0.0414 | -0.26689 | 0.77 | 0.63-0.92 | 0.00627 |
| **History of chronic constipation** |  |  |  |  |  |  |  |  |  |  |
| **No** | 28152 | 84.11 | Reference |  |  |  |  |  |  |  |
| **Yes** | 5317 | 15.89 | -0.43016 | 0.65 | 0.54-0.78 | 8.84E-06 | -0.49871 | 0.61 | 0.50-0.74 | 5.60E-07 |
| **History of bloody mucous stools** |  |  |  |  |  |  |  |  |  |  |
| **No** | 30057 | 89.81 | Reference |  |  |  |  |  |  |  |
| **Yes** | 3412 | 10.19 | 0.49736 | 1.64 | 1.39-1.94 | 4.83E-09 | 0.69186 | 2 | 1.66-2.39 | 6.23E-14 |
| **History of chronic appendicitis or appendectomy** |  |  |  |  |  |  |  |  |  |  |
| **No** | 31763 | 94.90 | Reference |  |  |  |  |  |  |  |
| **Yes** | 1706 | 5.10 | -0.01318 | 0.99 | 0.74-1.28 | 0.925 |  |  |  |  |
| **History of chronic cholecystitis or gallstones** |  |  |  |  |  |  |  |  |  |  |
| **No** | 32067 | 95.81 | Reference |  |  |  |  |  |  |  |
| **Yes** | 1402 | 4.19 | 0.18057 | 1.2 | 0.90-1.56 | 0.201 |  |  |  |  |
| **Adverse life events** |  |  |  |  |  |  |  |  |  |  |
| **No** | 31562 | 94.30 | Reference |  |  |  |  |  |  |  |
| **Yes** | 1907 | 5.70 | 0.04487 | 1.05 | 0.80-1.34 | 0.728 |  |  |  |  |
| **History of cancer** |  |  |  |  |  |  |  |  |  |  |
| **No** | 33128 | 98.98 | Reference |  |  |  |  |  |  |  |
| **Yes** | 341 | 1.02 | 0.14418 | 1.16 | 0.63-1.93 | 0.612 |  |  |  |  |
| **History of CRC in a first-degree relative** |  |  |  |  |  |  |  |  |  |  |
| **No** | 32307 | 96.53 | Reference |  |  |  |  |  |  |  |
| **Yes** | 1162 | 3.47 | 0.01085 | 1.01 | 0.72-1.38 | 0.948 |  |  |  |  |

NOTE: The number of participants in this logistic regression was 33469.

**Supplemental Table 7-1. Time to colonoscopy after a negative FIT and the adjusted incidence of CRC.**

|  |  | | **Unadjusted** | | | | **Adjusted** | | | |
| --- | --- | --- | --- | --- | --- | --- | --- | --- | --- | --- |
|  | **n** | **%** | **Beta** | **OR** | **95%CI** | **p Value** | **Beta** | **OR** | **95%CI** | **p Value** |
| **Time to Colonoscopy after FIT, mo** |  |  |  |  |  |  |  |  |  |  |
| **<1** | 4084 | 24.99 | Reference |  |  |  |  |  |  |  |
| **1-2** | 2488 | 15.23 | -1.169747 | 0.31 | 0.13-0.63 | 0.00248 | -1.19132 | 0.3 | 0.13-0.61 | 0.00209 |
| **2-3** | 1734 | 10.61 | -0.39951 | 0.67 | 0.34-1.24 | 0.224049 | -0.45459 | 0.63 | 0.32-1.18 | 0.16891 |
| **3-4** | 1181 | 7.23 | -0.893611 | 0.41 | 0.14-0.94 | 0.059526 | -1.04532 | 0.35 | 0.12-0.81 | 0.0281 |
| **4-5** | 679 | 4.16 | 0.002477 | 1 | 0.41-2.10 | 0.995184 | -0.13875 | 0.87 | 0.36-1.83 | 0.73614 |
| **5-6** | 406 | 2.48 | -0.333499 | 0.72 | 0.17-1.98 | 0.578258 | -0.49037 | 0.61 | 0.15-1.69 | 0.41444 |
| **6-12** | 1181 | 7.23 | 0.143691 | 1.15 | 0.61-2.07 | 0.64341 | -0.04016 | 0.96 | 0.50-1.73 | 0.89767 |
| **12-24** | 1287 | 7.88 | 0.685261 | 1.98 | 1.20-3.23 | 0.006461 | 0.59956 | 1.82 | 1.10-2.97 | 0.01782 |
| **24-36** | 1016 | 6.22 | 0.886987 | 2.43 | 1.45-3.97 | 0.000507 | 0.72458 | 2.06 | 1.23-3.39 | 0.0048 |
| **>36** | 2285 | 13.98 | 1.16969 | 3.22 | 2.21-4.76 | 1.99E-09 | 1.08422 | 2.96 | 2.02-4.38 | 3.44E-08 |
| **Gender** |  |  |  |  |  |  |  |  |  |  |
| **Female** | 9896 | 60.56 |  |  |  |  |  |  |  |  |
| **Male** | 6445 | 39.44 | 0.52871 | 1.7 | 1.30-2.22 | 0.000116 | 0.54354 | 1.72 | 1.31-2.26 | 8.67E-05 |
| **Age** |  |  |  |  |  |  |  |  |  |  |
| **40-59** | 7922 | 48.48 | Reference |  |  |  |  |  |  |  |
| **60-** | 8419 | 51.52 | 0.9058 | 2.47 | 1.84-3.36 | 3.01E-09 | 0.85513 | 2.35 | 1.75-3.21 | 3.53E-08 |
| **Education** |  |  |  |  |  |  |  |  |  |  |
| **Elementary school above** | 12998 | 79.54 | Reference |  |  |  |  |  |  |  |
| **Elementary School/below** | 3343 | 20.46 | 0.13359 | 1.14 | 0.82-1.56 | 0.414 |  |  |  |  |
| **Occupation** |  |  |  |  |  |  |  |  |  |  |
| **mental work** | 5255 | 32.16 | Reference |  |  |  |  |  |  |  |
| **manual work** | 11086 | 67.84 | 0.1426 | 1.15 | 0.86-1.56 | 0.344 |  |  |  |  |
| **Residential area** |  |  |  |  |  |  |  |  |  |  |
| **central urban** | 7170 | 43.88 | Reference |  |  |  |  |  |  |  |
| **agriculture-related areas** | 9171 | 56.12 | -0.53284 | 0.59 | 0.45-0.77 | 0.000117 | -0.27633 | 0.76 | 0.57-1.00 | 0.05127 |
| **History of chronic diarrhea** |  |  |  |  |  |  |  |  |  |  |
| **No** | 9917 | 60.69 | Reference |  |  |  |  |  |  |  |
| **Yes** | 6424 | 39.31 | -0.15852 | 0.85 | 0.64-1.13 | 0.268 |  |  |  |  |
| **History of chronic constipation** |  |  |  |  |  |  |  |  |  |  |
| **No** | 10090 | 61.75 | Reference |  |  |  |  |  |  |  |
| **Yes** | 6251 | 38.25 | 0.10552 | 1.11 | 0.84-1.46 | 0.449 |  |  |  |  |
| **History of bloody mucous stools** |  |  |  |  |  |  |  |  |  |  |
| **No** | 11473 | 70.21 | Reference |  |  |  |  |  |  |  |
| **Yes** | 4868 | 29.79 | 0.01461 | 1.01 | 0.75-1.35 | 0.922 |  |  |  |  |
| **History of chronic appendicitis or appendectomy** |  |  |  |  |  |  |  |  |  |  |
| **No** | 13127 | 80.33 | Reference |  |  |  |  |  |  |  |
| **Yes** | 3214 | 19.67 | -0.23893 | 0.79 | 0.54-1.12 | 0.198 |  |  |  |  |
| **History of chronic cholecystitis or gallstones** |  |  |  |  |  |  |  |  |  |  |
| **No** | 13172 | 80.61 | Reference |  |  |  |  |  |  |  |
| **Yes** | 3169 | 19.39 | 0.09091 | 1.1 | 0.78-1.51 | 0.59 |  |  |  |  |
| **Adverse life events** |  |  |  |  |  |  |  |  |  |  |
| **No** | 12389 | 75.82 | Reference |  |  |  |  |  |  |  |
| **Yes** | 3952 | 24.18 | 0.1897 | 1.21 | 0.89-1.62 | 0.215 |  |  |  |  |
| **History of cancer** |  |  |  |  |  |  |  |  |  |  |
| **No** | 15061 | 92.17 | Reference |  |  |  |  |  |  |  |
| **Yes** | 1280 | 7.83 | 0.29281 | 1.34 | 0.84-2.04 | 0.197 |  |  |  |  |
| **History of CRC in a first-degree relative** |  |  |  |  |  |  |  |  |  |  |
| **No** | 12362 | 75.65 | Reference |  |  |  |  |  |  |  |
| **Yes** | 3979 | 24.35 | -0.12071 | 0.89 | 0.64-1.21 | 0.464 |  |  |  |  |

NOTE: The number of participants in this logistic regression was 16341.

**Supplemental Table 7-2. Time to colonoscopy after a negative FIT and the adjusted incidence of advanced neoplasia.**

|  |  | | Unadjusted | | | | Adjusted | | | |
| --- | --- | --- | --- | --- | --- | --- | --- | --- | --- | --- |
|  | n | % | Beta | OR | 95%CI | p Value | Beta | OR | 95%CI | p Value |
| **Time to Colonoscopy after FIT, mo** |  |  |  |  |  |  |  |  |  |  |
| **<1** | 4084 | 24.99 |  |  |  |  |  |  |  |  |
| **1-2** | 2488 | 15.23 | -0.30579 | 0.74 | 0.56-0.98 | 0.033038 | -0.301933 | 0.74 | 0.56-0.98 | 0.03665 |
| **2-3** | 1734 | 10.61 | -0.13542 | 0.87 | 0.65-1.18 | 0.379356 | -0.146992 | 0.86 | 0.64-1.17 | 0.345439 |
| **3-4** | 1181 | 7.23 | -0.08262 | 0.92 | 0.65-1.30 | 0.636711 | -0.169817 | 0.84 | 0.60-1.19 | 0.337757 |
| **4-5** | 679 | 4.16 | 0.11897 | 1.13 | 0.76-1.68 | 0.558454 | 0.01321 | 1.01 | 0.68-1.51 | 0.948649 |
| **5-6** | 406 | 2.48 | 0.2331 | 1.26 | 0.78-2.03 | 0.337455 | 0.093496 | 1.1 | 0.68-1.77 | 0.702622 |
| **6-12** | 1181 | 7.23 | 0.17411 | 1.19 | 0.87-1.63 | 0.2759 | 0.006683 | 1.01 | 0.73-1.38 | 0.967022 |
| **12-24** | 1287 | 7.88 | 0.47934 | 1.62 | 1.23-2.13 | 0.000657 | 0.412422 | 1.51 | 1.14-2.00 | 0.00374 |
| **24-36** | 1016 | 6.22 | 0.64927 | 1.91 | 1.44-2.54 | 7.61E-06 | 0.510735 | 1.67 | 1.25-2.22 | 0.000505 |
| **>36** | 2285 | 13.98 | 0.89734 | 2.45 | 1.98-3.03 | < 2e-16 | 0.837282 | 2.31 | 1.86-2.86 | 2.36E-14 |
| **Gender** |  |  |  |  |  |  |  |  |  |  |
| **Female** | 9896 | 60.56 |  |  |  |  |  |  |  |  |
| **Male** | 6445 | 39.44 | 0.6647 | 1.94 | 1.69-2.24 | <2e-16 | 0.68727 | 1.99 | 1.72-2.30 | < 2e-16 |
| **Age** |  |  |  |  |  |  |  |  |  |  |
| **40-59** | 7922 | 48.48 |  |  |  |  |  |  |  |  |
| **60-** | 8419 | 51.52 | 0.69505 | 2 | 1.72-2.33 | <2e-16 | 0.631483 | 1.88 | 1.61-2.20 | 1.92E-15 |
| **Education** |  |  |  |  |  |  |  |  |  |  |
| **Elementary school above** | 12998 | 79.54 |  |  |  |  |  |  |  |  |
| **Elementary School/below** | 3343 | 20.46 | -0.20995 | 0.81 | 0.67-0.98 | 0.0272 | 0.042553 | 1.04 | 0.85-1.29 | 0.691696 |
| **Occupation** |  |  |  |  |  |  |  |  |  |  |
| **mental work** | 5255 | 32.16 |  |  |  |  |  |  |  |  |
| **manual work** | 11086 | 67.84 | -0.000749 | 1 | 0.86-1.16 | 0.992 |  |  |  |  |
| **Residential area** |  |  |  |  |  |  |  |  |  |  |
| **central urban** | 7170 | 43.88 |  |  |  |  |  |  |  |  |
| **agriculture-related areas** | 9171 | 56.12 | -0.54047 | 0.58 | 0.50-0.67 | 1.27E-13 | -0.401164 | 0.67 | 0.57-0.79 | 1.43E-06 |
| **History of chronic diarrhea** |  |  |  |  |  |  |  |  |  |  |
| **No** | 9917 | 60.69 |  |  |  |  |  |  |  |  |
| **Yes** | 6424 | 39.31 | -0.13591 | 0.87 | 0.75-1.01 | 0.0704 |  |  |  |  |
| **History of chronic constipation** |  |  |  |  |  |  |  |  |  |  |
| **No** | 10090 | 61.75 |  |  |  |  |  |  |  |  |
| **Yes** | 6251 | 38.25 | -0.12878 | 0.88 | 0.76-1.02 | 0.0881 |  |  |  |  |
| **History of bloody mucous stools** |  |  |  |  |  |  |  |  |  |  |
| **No** | 11473 | 70.21 |  |  |  |  |  |  |  |  |
| **Yes** | 4868 | 29.79 | -0.12676 | 0.88 | 0.75-1.03 | 0.118 |  |  |  |  |
| **History of chronic appendicitis or appendectomy** |  |  |  |  |  |  |  |  |  |  |
| **No** | 13127 | 80.33 |  |  |  |  |  |  |  |  |
| **Yes** | 3214 | 19.67 | -0.08752 | 0.92 | 0.76-1.10 | 0.348 |  |  |  |  |
| **History of chronic cholecystitis or gallstones** |  |  |  |  |  |  |  |  |  |  |
| **No** | 13172 | 80.61 |  |  |  |  |  |  |  |  |
| **Yes** | 3169 | 19.39 | 0.15924 | 1.17 | 0.99-1.39 | 0.0693 |  |  |  |  |
| **Adverse life events** |  |  |  |  |  |  |  |  |  |  |
| **No** | 12389 | 75.82 |  |  |  |  |  |  |  |  |
| **Yes** | 3952 | 24.18 | -0.12242 | 0.88 | 0.75-1.05 | 0.159 |  |  |  |  |
| **History of cancer** |  |  |  |  |  |  |  |  |  |  |
| **No** | 15061 | 92.17 |  |  |  |  |  |  |  |  |
| **Yes** | 1280 | 7.83 | 0.12008 | 1.13 | 0.88-1.45 | 0.35 |  |  |  |  |
| **History of CRC in a first-degree relative** |  |  |  |  |  |  |  |  |  |  |
| **No** | 12362 | 75.65 |  |  |  |  |  |  |  |  |
| **Yes** | 3979 | 24.35 | 0.27391 | 1.32 | 1.13-1.54 | 0.000577 | 0.230269 | 1.26 | 1.07-1.48 | 0.004785 |

NOTE: The number of participants in this logistic regression was 16341.

**Supplemental Table 8. Time to colonoscopy after a positive HRFQ and the adjusted incidence of CRC.**

|  |  |  | **Unadjusted** | | | | **Adjusted** | | | |
| --- | --- | --- | --- | --- | --- | --- | --- | --- | --- | --- |
|  | **n** | **%** | **Beta** | **OR** | **95%CI** | **p Value** | **Beta** | **OR** | **95%CI** | **p Value** |
| **Time to Colonoscopy after FIT, mo** |  |  |  |  |  |  |  |  |  |  |
| **<1** | 6736 | 30.74 | Reference |  |  |  |  |  |  |  |
| **1-2** | 3482 | 15.89 | -0.9771 | 0.38 | 0.24-0.56 | 5.82E-06 | -0.92314 | 0.4 | 0.25-0.60 | 2.13E-05 |
| **2-3** | 2359 | 10.76 | -0.5092 | 0.6 | 0.39-0.89 | 0.01505 | -0.44891 | 0.64 | 0.41-0.95 | 0.03441 |
| **3-4** | 1502 | 6.85 | -0.7535 | 0.47 | 0.26-0.79 | 0.00765 | -0.68906 | 0.5 | 0.27-0.85 | 0.01596 |
| **4-5** | 836 | 3.81 | -0.16 | 0.85 | 0.47-1.43 | 0.57237 | -0.03663 | 0.96 | 0.53-1.64 | 0.89863 |
| **5-6** | 498 | 2.27 | -0.6784 | 0.51 | 0.18-1.12 | 0.13851 | -0.54712 | 0.58 | 0.20-1.29 | 0.23562 |
| **6-12** | 1403 | 6.40 | -0.1817 | 0.83 | 0.52-1.28 | 0.42521 | -0.01765 | 0.98 | 0.61-1.52 | 0.93959 |
| **12-24** | 1445 | 6.59 | 0.1564 | 1.17 | 0.78-1.70 | 0.4269 | 0.51549 | 1.67 | 1.11-2.47 | 0.01179 |
| **24-36** | 1119 | 5.11 | 0.3208 | 1.38 | 0.91-2.03 | 0.1174 | 0.62328 | 1.87 | 1.21-2.80 | 0.00355 |
| **>36** | 2535 | 11.57 | 0.5991 | 1.82 | 1.38-2.39 | 1.68E-05 | 1.0089 | 2.74 | 2.04-3.68 | 2.10E-11 |
| **Gender** |  |  |  |  |  |  |  |  |  |  |
| **Female** | 12925 | 58.98 | Reference |  |  |  |  |  |  |  |
| **Male** | 8990 | 41.02 | 0.66144 | 1.94 | 1.59-2.37 | 1.21E-10 | 0.59957 | 1.82 | 1.49-2.24 | 9.39E-09 |
| **Age** |  |  |  |  |  |  |  |  |  |  |
| **40-59** | 10661 | 48.65 | Reference |  |  |  |  |  |  |  |
| **60-** | 11254 | 51.35 | 0.98766 | 2.68 | 2.15-3.38 | <2e-16 | 1.00968 | 2.74 | 2.19-3.47 | < 2e-16 |
| **Education** |  |  |  |  |  |  |  |  |  |  |
| **Elementary school above** | 17385 | 79.33 | Reference |  |  |  |  |  |  |  |
| **Elementary School/below** | 4530 | 20.67 | 0.08552 | 1.09 | 0.85-1.38 | 0.485 |  |  |  |  |
| **Occupation** |  |  |  |  |  |  |  |  |  |  |
| **mental work** | 6927 | 31.61 | Reference |  |  |  |  |  |  |  |
| **manual work** | 14988 | 68.39 | 0.06701 | 1.07 | 0.86-1.33 | 0.545 |  |  |  |  |
| **Residential area** |  |  |  |  |  |  |  |  |  |  |
| **central urban** | 9651 | 44.04 | Reference |  |  |  |  |  |  |  |
| **agriculture-related areas** | 12264 | 55.96 | -0.46201 | 0.63 | 0.52-0.77 | 6.25E-06 | -0.2877 | 0.75 | 0.61-0.92 | 0.00592 |
| **FIT** |  |  |  |  |  |  |  |  |  |  |
| **negative** | 16341 | 74.57 | Reference |  |  |  |  |  |  |  |
| **positive** | 5574 | 25.43 | 0.9012 | 2.46 | 2.01-3.01 | <2e-16 | 1.08294 | 2.95 | 2.37-3.68 | < 2e-16 |
| **History of chronic diarrhea** |  |  |  |  |  |  |  |  |  |  |
| **No** | 12929 | 59.00 | Reference |  |  |  |  |  |  |  |
| **Yes** | 8986 | 41.00 | -0.01664 | 0.98 | 0.80-1.20 | 0.872 |  |  |  |  |
| **History of chronic constipation** |  |  |  |  |  |  |  |  |  |  |
| **No** | 13213 | 60.29 | Reference |  |  |  |  |  |  |  |
| **Yes** | 8702 | 39.71 | -0.12529 | 0.88 | 0.72-1.08 | 0.234 |  |  |  |  |
| **History of bloody mucous stools** |  |  |  |  |  |  |  |  |  |  |
| **No** | 14575 | 66.51 | Reference |  |  |  |  |  |  |  |
| **Yes** | 7340 | 33.49 | 0.37572 | 1.46 | 1.19-1.78 | 0.000266 | 0.33493 | 1.4 | 1.13-1.72 | 0.00182 |
| **History of chronic appendicitis or appendectomy** |  |  |  |  |  |  |  |  |  |  |
| **No** | 17779 | 81.13 | Reference |  |  |  |  |  |  |  |
| **Yes** | 4136 | 18.87 | -0.24303 | 0.78 | 0.59-1.02 | 0.0834 |  |  |  |  |
| **History of chronic cholecystitis or gallstones** |  |  |  |  |  |  |  |  |  |  |
| **No** | 17900 | 81.68 | Reference |  |  |  |  |  |  |  |
| **Yes** | 4015 | 18.32 | 0.09754 | 1.1 | 0.85-1.41 | 0.445 |  |  |  |  |
| **Adverse life events** |  |  |  |  |  |  |  |  |  |  |
| **No** | 16931 | 77.26 | Reference |  |  |  |  |  |  |  |
| **Yes** | 4984 | 22.74 | 0.06359 | 1.07 | 0.84-1.34 | 0.594 |  |  |  |  |
| **History of cancer** |  |  |  |  |  |  |  |  |  |  |
| **No** | 20294 | 92.60 | Reference |  |  |  |  |  |  |  |
| **Yes** | 1621 | 7.40 | 0.20329 | 1.23 | 0.85-1.71 | 0.256 |  |  |  |  |
| **History of CRC in a first-degree relative** |  |  |  |  |  |  |  |  |  |  |
| **No** | 16774 | 76.54 | Reference |  |  |  |  |  |  |  |
| **Yes** | 5141 | 23.46 | -0.07976 | 0.92 | 0.72-1.17 | 0.515 |  |  |  |  |

NOTE: The number of participants in this logistic regression was 21915.

**Supplemental Table 9. Time to colonoscopy after a negative HRFQ and the adjusted incidence of CRC.**

|  |  |  | **Unadjusted** | | | | **Adjusted** | | | |
| --- | --- | --- | --- | --- | --- | --- | --- | --- | --- | --- |
|  | **n** | **%** | **Beta** | **OR** | **95%CI** | **p Value** | **Beta** | **OR** | **95%CI** | **p Value** |
| **Time to Colonoscopy after FIT, mo** |  |  |  |  |  |  |  |  |  |  |
| **<1** | 12895 | 46.23 | Reference |  |  |  |  |  |  |  |
| **1-2** | 5275 | 18.91 | 0.04143 | 1.04 | 0.85-1.27 | 0.6875 | -0.04616 | 0.95 | 0.78-1.17 | 0.65665 |
| **2-3** | 3088 | 11.07 | -0.06249 | 0.94 | 0.72-1.21 | 0.6337 | -0.21395 | 0.81 | 0.62-1.04 | 0.10574 |
| **3-4** | 1546 | 5.54 | 0.28035 | 1.32 | 0.97-1.77 | 0.0671 | 0.12424 | 1.13 | 0.83-1.52 | 0.42131 |
| **4-5** | 865 | 3.10 | -0.03523 | 0.97 | 0.60-1.47 | 0.8772 | -0.19734 | 0.82 | 0.51-1.25 | 0.38937 |
| **5-6** | 548 | 1.96 | 0.48415 | 1.62 | 1.02-2.46 | 0.0312 | 0.29949 | 1.35 | 0.84-2.05 | 0.18598 |
| **6-12** | 1050 | 3.76 | 0.95643 | 2.6 | 1.97-3.39 | 6.05E-12 | 0.76421 | 2.15 | 1.62-2.81 | 5.90E-08 |
| **12-24** | 949 | 3.40 | 1.12811 | 3.09 | 2.35-4.01 | < 2e-16 | 1.02086 | 2.78 | 2.10-3.62 | 1.51E-13 |
| **24-36** | 620 | 2.22 | 1.42481 | 4.16 | 3.09-5.50 | < 2e-16 | 1.31824 | 3.74 | 2.77-4.97 | < 2e-16 |
| **>36** | 1059 | 3.80 | 1.49329 | 4.45 | 3.54-5.57 | < 2e-16 | 1.36946 | 3.93 | 3.11-4.94 | < 2e-16 |
| **Gender** |  |  |  |  |  |  |  |  |  |  |
| **Female** | 14230 | 51.01 | Reference |  |  |  |  |  |  |  |
| **Male** | 13665 | 48.99 | 0.27775 | 1.32 | 1.16-1.51 | 3.38E-05 | 0.1985 | 1.22 | 1.07-1.40 | 0.00396 |
| **Age** |  |  |  |  |  |  |  |  |  |  |
| **40-59** | 12237 | 43.87 | Reference |  |  |  |  |  |  |  |
| **60-** | 15658 | 56.13 | 1.11144 | 3.04 | 2.60-3.57 | <2e-16 | 0.99783 | 2.71 | 2.30-3.21 | < 2e-16 |
| **Education** |  |  |  |  |  |  |  |  |  |  |
| **Elementary school above** | 20263 | 72.64 | Reference |  |  |  |  |  |  |  |
| **Elementary School/below** | 7632 | 27.36 | -0.21973 | 0.8 | 0.69-0.93 | 0.00515 | -0.06161 | 0.94 | 0.79-1.12 | 0.49961 |
| **Occupation** |  |  |  |  |  |  |  |  |  |  |
| **mental work** | 7491 | 26.85 | Reference |  |  |  |  |  |  |  |
| **manual work** | 20404 | 73.15 | -0.05121 | 0.95 | 0.82-1.10 | 0.491 |  |  |  |  |
| **Residential area** |  |  |  |  |  |  |  |  |  |  |
| **central urban** | 8873 | 31.81 | Reference |  |  |  |  |  |  |  |
| **agriculture-related areas** | 19022 | 68.19 | -0.76952 | 0.46 | 0.41-0.53 | <2e-16 | -0.5521 | 0.58 | 0.49-0.67 | 1.19E-12 |
| **History of chronic diarrhea** |  |  |  |  |  |  |  |  |  |  |
| **No** | 25561 | 91.63 | Reference |  |  |  |  |  |  |  |
| **Yes** | 2334 | 8.37 | -0.36948 | 0.69 | 0.52-0.90 | 0.00811 | -0.19155 | 0.83 | 0.62-1.08 | 0.17748 |
| **History of chronic constipation** |  |  |  |  |  |  |  |  |  |  |
| **No** | 25029 | 89.73 | Reference |  |  |  |  |  |  |  |
| **Yes** | 2866 | 10.27 | -0.42142 | 0.66 | 0.50-0.84 | 0.00111 | -0.35298 | 0.7 | 0.54-0.90 | 0.00735 |
| **History of bloody mucous stools** |  |  |  |  |  |  |  |  |  |  |
| **No** | 26955 | 96.63 | Reference |  |  |  |  |  |  |  |
| **Yes** | 940 | 3.37 | 0.9043 | 2.47 | 1.91-3.15 | 1.73E-12 | 0.84717 | 2.33 | 1.79-3.01 | 1.69E-10 |
| **History of chronic appendicitis or appendectomy** |  |  |  |  |  |  |  |  |  |  |
| **No** | 27111 | 97.19 | Reference |  |  |  |  |  |  |  |
| **Yes** | 784 | 2.81 | 0.14276 | 1.15 | 0.78-1.64 | 0.451 |  |  |  |  |
| **History of chronic cholecystitis or gallstones** |  |  |  |  |  |  |  |  |  |  |
| **No** | 27339 | 98.01 | Reference |  |  |  |  |  |  |  |
| **Yes** | 556 | 1.99 | 0.17715 | 1.19 | 0.75-1.79 | 0.421 |  |  |  |  |
| **Adverse life events** |  |  |  |  |  |  |  |  |  |  |
| **No** | 27020 | 96.86 | Reference |  |  |  |  |  |  |  |
| **Yes** | 875 | 3.14 | 0.09453 | 1.1 | 0.75-1.55 | 0.606 |  |  |  |  |

NOTE: The number of participants in this logistic regression was 27895.

**Supplemental Table 10. Time to colonoscopy after a positive HRFQ and positive FIT and the adjusted incidence of CRC.**

|  |  |  | **Unadjusted** | | | | **Adjusted** | | | |
| --- | --- | --- | --- | --- | --- | --- | --- | --- | --- | --- |
|  | **n** | **%** | **Beta** | **OR** | **95%CI** | **p Value** | **Beta** | **OR** | **95%CI** | **p Value** |
| **Time to Colonoscopy after FIT, mo** |  |  |  |  |  |  |  |  |  |  |
| **<1** | 2652 | 47.58 | Reference |  |  |  |  |  |  |  |
| **1-2** | 994 | 17.83 | -0.6444 | 0.53 | 0.31-0.85 | 0.0135 | -0.76768 | 0.46 | 0.27-0.76 | 0.003605 |
| **2-3** | 625 | 11.21 | -0.2905 | 0.75 | 0.42-1.25 | 0.2909 | -0.45546 | 0.63 | 0.35-1.07 | 0.102395 |
| **3-4** | 321 | 5.76 | -0.197 | 0.82 | 0.38-1.56 | 0.5785 | -0.39466 | 0.67 | 0.31-1.29 | 0.272605 |
| **4-5** | 157 | 2.82 | 0.284 | 1.33 | 0.55-2.72 | 0.4791 | 0.12154 | 1.13 | 0.46-2.34 | 0.765264 |
| **5-6** | 92 | 1.65 | -0.4579 | 0.63 | 0.10-2.05 | 0.5264 | -0.6101 | 0.54 | 0.09-1.78 | 0.401454 |
| **6-12** | 222 | 3.98 | 0.1847 | 1.2 | 0.56-2.29 | 0.6048 | 0.04821 | 1.05 | 0.48-2.02 | 0.893904 |
| **12-24** | 158 | 2.83 | 0.2774 | 1.32 | 0.55-2.70 | 0.4894 | 0.23971 | 1.27 | 0.52-2.65 | 0.557286 |
| **24-36** | 103 | 1.85 | 0.3732 | 1.45 | 0.50-3.31 | 0.428 | 0.21962 | 1.25 | 0.43-2.89 | 0.645911 |
| **>36** | 250 | 4.49 | 0.5972 | 1.82 | 1.00-3.10 | 0.0375 | 0.69949 | 2.01 | 1.09-3.47 | 0.016895 |
| **Gender** |  |  |  |  |  |  |  |  |  |  |
| **Female** | 3029 | 54.34 | Reference |  |  |  |  |  |  |  |
| **Male** | 2545 | 45.66 | 0.7259 | 2.07 | 1.52-2.83 | 4.08E-06 | 0.61402 | 1.85 | 1.35-2.55 | 0.000148 |
| **Age** |  |  |  |  |  |  |  |  |  |  |
| **40-59** | 2739 | 49.14 | Reference |  |  |  |  |  |  |  |
| **60-** | 2835 | 50.86 | 1.1114 | 3.04 | 2.17-4.33 | 2.60E-10 | 1.18584 | 3.27 | 2.32-4.71 | 5.14E-11 |
| **Education** |  |  |  |  |  |  |  |  |  |  |
| **Elementary school above** | 4387 | 78.70 | Reference |  |  |  |  |  |  |  |
| **Elementary School/below** | 1187 | 21.30 | 0.003265 | 1 | 0.69-1.43 | 0.986 |  |  |  |  |
| **Occupation** |  |  |  |  |  |  |  |  |  |  |
| **mental work** | 1672 | 30.00 | Reference |  |  |  |  |  |  |  |
| **manual work** | 3902 | 70.00 | -0.0711 | 0.93 | 0.68-1.29 | 0.665 |  |  |  |  |
| **Residential area** |  |  |  |  |  |  |  |  |  |  |
| **central urban** | 2481 | 44.51 | Reference |  |  |  |  |  |  |  |
| **agriculture-related areas** | 3093 | 55.49 | -0.3674 | 0.69 | 0.51-0.93 | 0.0162 | -0.28211 | 0.75 | 0.56-1.02 | 0.070017 |
| **History of chronic diarrhea** |  |  |  |  |  |  |  |  |  |  |
| **No** | 3012 | 54.04 | Reference |  |  |  |  |  |  |  |
| **Yes** | 2562 | 45.96 | 0.02766 | 1.03 | 0.76-1.39 | 0.856 |  |  |  |  |
| **History of chronic constipation** |  |  |  |  |  |  |  |  |  |  |
| **No** | 3123 | 56.03 | Reference |  |  |  |  |  |  |  |
| **Yes** | 2451 | 43.97 | -0.52643 | 0.59 | 0.43-0.81 | 0.00124 | -0.45279 | 0.64 | 0.46-0.88 | 0.006447 |
| **History of bloody mucous stools** |  |  |  |  |  |  |  |  |  |  |
| **No** | 3102 | 55.65 | Reference |  |  |  |  |  |  |  |
| **Yes** | 2472 | 44.35 | 0.5149 | 1.67 | 1.24-2.27 | 0.000811 | 0.53768 | 1.71 | 1.26-2.34 | 0.00066 |
| **History of chronic appendicitis or appendectomy** |  |  |  |  |  |  |  |  |  |  |
| **No** | 4652 | 83.46 | Reference |  |  |  |  |  |  |  |
| **Yes** | 922 | 16.54 | -0.15189 | 0.86 | 0.55-1.29 | 0.481 |  |  |  |  |
| **History of chronic cholecystitis or gallstones** |  |  |  |  |  |  |  |  |  |  |
| **No** | 4728 | 84.82 | Reference |  |  |  |  |  |  |  |
| **Yes** | 846 | 15.18 | 0.24915 | 1.28 | 0.86-1.86 | 0.205 |  |  |  |  |
| **Adverse life events** |  |  |  |  |  |  |  |  |  |  |
| **No** | 4542 | 81.49 | Reference |  |  |  |  |  |  |  |
| **Yes** | 1032 | 18.51 | 0.0397 | 1.04 | 0.70-1.50 | 0.838 |  |  |  |  |
| **History of cancer** |  |  |  |  |  |  |  |  |  |  |
| **No** | 5233 | 93.88 | Reference |  |  |  |  |  |  |  |
| **Yes** | 341 | 6.12 | 0.19669 | 1.22 | 0.65-2.08 | 0.503 |  |  |  |  |
| **History of CRC in a first-degree relative** |  |  |  |  |  |  |  |  |  |  |
| **No** | 4412 | 79.15 | Reference |  |  |  |  |  |  |  |
| **Yes** | 1162 | 20.85 | 0.0654 | 1.07 | 0.73-1.52 | 0.723 |  |  |  |  |

NOTE: The number of participants in this logistic regression was 5574.

**Supplemental Table 11. Time to colonoscopy after a FIT and the adjusted incidence of CRC: sensitivity analyses.**

**① Reference group: 8-30 days**

|  |  | | **Unadjusted** | | | | **Adjusted** | | | |
| --- | --- | --- | --- | --- | --- | --- | --- | --- | --- | --- |
|  | **n** | **%** | **Beta** | **OR** | **95%CI** | **p Value** | **Beta** | **OR** | **95%CI** | **p Value** |
| **Time to Colonoscopy after FIT** |  |  |  |  |  |  |  |  |  |  |
| **8-30days** | 13612 | 31.08 | Reference |  |  |  |  |  |  |  |
| **1-2months** | 8757 | 20.00 | -0.2096 | 0.81 | 0.67-0.98 | 0.031368 | -0.234069 | 0.79 | 0.65-0.96 | 0.0172 |
| **2-3months** | 5447 | 12.44 | -0.21974 | 0.8 | 0.64-1.00 | 0.057247 | -0.2560304 | 0.77 | 0.61-0.97 | 0.0284 |
| **3-4months** | 3048 | 6.96 | -0.07706 | 0.93 | 0.70-1.20 | 0.576029 | -0.0743874 | 0.93 | 0.70-1.21 | 0.5939 |
| **4-5months** | 1701 | 3.88 | -0.11359 | 0.89 | 0.62-1.25 | 0.528244 | -0.0941581 | 0.91 | 0.63-1.28 | 0.6045 |
| **5-6months** | 1046 | 2.39 | 0.1185 | 1.13 | 0.74-1.64 | 0.559765 | 0.0956838 | 1.1 | 0.72-1.61 | 0.6409 |
| **6-12months** | 2453 | 5.60 | 0.46977 | 1.6 | 1.25-2.02 | 0.000121 | 0.5553796 | 1.74 | 1.36-2.21 | 8.37E-06 |
| **12-24months** | 2394 | 5.47 | 0.64723 | 1.91 | 1.52-2.39 | 2.30E-08 | 0.8734726 | 2.4 | 1.89-3.01 | 1.97E-13 |
| **24-36months** | 1739 | 3.97 | 0.84113 | 2.32 | 1.81-2.93 | 6.39E-12 | 1.1061534 | 3.02 | 2.35-3.86 | < 2e-16 |
| **>36months** | 3594 | 8.21 | 0.90715 | 2.48 | 2.06-2.97 | < 2e-16 | 1.2892124 | 3.63 | 2.99-4.39 | < 2e-16 |
| **Gender** |  |  |  |  |  |  |  |  |  |  |
| **Female** | 24047 | 54.91 | Reference |  |  |  |  |  |  |  |
| **Male** | 19744 | 45.09 | 0.41686 | 1.52 | 1.35-1.70 | 1.93E-12 | 0.3104375 | 1.36 | 1.21-1.54 | 3.36E-07 |
| **Age** |  |  |  |  |  |  |  |  |  |  |
| **40-59** | 19999 | 45.67 | Reference |  |  |  |  |  |  |  |
| **60-** | 23792 | 54.33 | 1.08719 | 2.97 | 2.59-3.41 | <2e-16 | 0.9546989 | 2.6 | 2.26-3.00 | < 2e-16 |
| **Education** |  |  |  |  |  |  |  |  |  |  |
| **Elementary school above** | 33221 | 75.86 | Reference |  |  |  |  |  |  |  |
| **Elementary School/below** | 10570 | 24.14 | -0.06757 | 0.93 | 0.81-1.07 | 0.334 |  |  |  |  |
| **Occupation** |  |  |  |  |  |  |  |  |  |  |
| **mental work** | 12718 | 29.04 | Reference |  |  |  |  |  |  |  |
| **manual work** | 31073 | 70.96 | 0.009081 | 1.01 | 0.89-1.15 | 0.889 |  |  |  |  |
| **Residential area** |  |  |  |  |  |  |  |  |  |  |
| **central urban** | 16755 | 38.26 | Reference |  |  |  |  |  |  |  |
| **agriculture-related areas** | 27036 | 61.74 | -0.55315 | 0.58 | 0.51-0.65 | <2e-16 | -0.4791551 | 0.62 | 0.55-0.70 | 3.41E-15 |
| **FIT** |  |  |  |  |  |  |  |  |  |  |
| **Negative** | 14965 | 34.17 | Reference |  |  |  |  |  |  |  |
| **Positive** | 28826 | 65.83 | 0.97236 | 2.64 | 2.27-3.09 | <2e-16 | 1.1646768 | 3.2 | 2.64-3.90 | < 2e-16 |
| **History of chronic diarrhea** |  |  |  |  |  |  |  |  |  |  |
| **No** | 33950 | 77.53 | Reference |  |  |  |  |  |  |  |
| **Yes** | 9841 | 22.47 | -0.52685 | 0.59 | 0.50-0.69 | 1.87E-10 | -0.1895603 | 0.83 | 0.70-0.98 | 0.029 |
| **History of chronic constipation** |  |  |  |  |  |  |  |  |  |  |
| **No** | 33548 | 76.61 | Reference |  |  |  |  |  |  |  |
| **Yes** | 10243 | 23.39 | -0.47434 | 0.62 | 0.53-0.73 | 2.80E-09 | -0.2453987 | 0.78 | 0.66-0.92 | 0.0034 |
| **History of bloody mucous stools** |  |  |  |  |  |  |  |  |  |  |
| **No** | 36606 | 83.59 | Reference |  |  |  |  |  |  |  |
| **Yes** | 7185 | 16.41 | -0.02183 | 0.98 | 0.83-1.14 | 0.785 |  |  |  |  |
| **History of chronic appendicitis or appendectomy** |  |  |  |  |  |  |  |  |  |  |
| **No** | 39303 | 89.75 | Reference |  |  |  |  |  |  |  |
| **Yes** | 4488 | 10.25 | -0.4392 | 0.64 | 0.51-0.80 | 0.000144 | -0.214868 | 0.81 | 0.63-1.01 | 0.073 |
| **History of chronic cholecystitis or gallstones** |  |  |  |  |  |  |  |  |  |  |
| **No** | 39620 | 90.48 | Reference |  |  |  |  |  |  |  |
| **Yes** | 4171 | 9.52 | -0.21739 | 0.8 | 0.65-0.99 | 0.0466 | -0.0001327 | 1 | 0.79-1.25 | 0.9991 |
| **Adverse life events** |  |  |  |  |  |  |  |  |  |  |
| **No** | 38380 | 87.64 | Reference |  |  |  |  |  |  |  |
| **Yes** | 5411 | 12.36 | -0.25863 | 0.77 | 0.63-0.93 | 0.00863 | 0.0661953 | 1.07 | 0.87-1.31 | 0.5257 |
| **History of cancer** |  |  |  |  |  |  |  |  |  |  |
| **No** | 42289 | 96.57 | Reference |  |  |  |  |  |  |  |
| **Yes** | 1502 | 3.43 | -0.18899 | 0.83 | 0.58-1.15 | 0.283 |  |  |  |  |
| **History of CRC in a first-degree relative** |  |  |  |  |  |  |  |  |  |  |
| **No** | 39048 | 89.17 | Reference |  |  |  |  |  |  |  |
| **Yes** | 4743 | 10.83 | -0.5429 | 0.58 | 0.46-0.73 | 3.81E-06 | -0.1972044 | 0.82 | 0.63-1.05 | 0.1232 |

NOTE: The number of participants in this logistic regression was 43791.

**② Reference group: 8-30 days, and includes 1-7 days**

|  |  | | **Unadjusted** | | | | **Adjusted** | | | |
| --- | --- | --- | --- | --- | --- | --- | --- | --- | --- | --- |
|  | **n** | **%** | **Beta** | **OR** | **95%CI** | **p Value** | **Beta** | **OR** | **95%CI** | **p Value** |
| **Time to Colonoscopy after FIT** |  |  |  |  |  |  |  |  |  |  |
| **8-30 days** | 13612 | 28.78 | Reference |  |  |  |  |  |  |  |
| **1-7days** | 3503 | 7.41 | 0.09064 | 1.09 | 0.86-1.38 | 0.458123 | 0.120497 | 1.13 | 0.88-1.43 | 0.32854 |
| **1-2months** | 8757 | 18.52 | -0.2096 | 0.81 | 0.67-0.98 | 0.031368 | -0.237489 | 0.79 | 0.65-0.95 | 0.01562 |
| **2-3months** | 5447 | 11.52 | -0.21974 | 0.8 | 0.64-1.00 | 0.057247 | -0.26099 | 0.77 | 0.61-0.96 | 0.02546 |
| **3-4months** | 3048 | 6.44 | -0.07706 | 0.93 | 0.70-1.20 | 0.576029 | -0.079211 | 0.92 | 0.70-1.21 | 0.57014 |
| **4-5months** | 1701 | 3.60 | -0.11359 | 0.89 | 0.62-1.25 | 0.528244 | -0.099151 | 0.91 | 0.62-1.27 | 0.58542 |
| **5-6months** | 1046 | 2.21 | 0.1185 | 1.13 | 0.74-1.64 | 0.559765 | 0.089462 | 1.09 | 0.72-1.60 | 0.66271 |
| **6-12months** | 2453 | 5.19 | 0.46977 | 1.6 | 1.25-2.02 | 0.000121 | 0.545206 | 1.72 | 1.34-2.19 | 1.22E-05 |
| **12-24months** | 2394 | 5.06 | 0.64723 | 1.91 | 1.52-2.39 | 2.30E-08 | 0.866049 | 2.38 | 1.88-2.99 | 3.14E-13 |
| **24-36months** | 1739 | 3.68 | 0.84113 | 2.32 | 1.81-2.93 | 6.39E-12 | 1.097574 | 3 | 2.33-3.82 | < 2e-16 |
| **>36months** | 3594 | 7.60 | 0.90715 | 2.48 | 2.06-2.97 | < 2e-16 | 1.278483 | 3.59 | 2.96-4.35 | < 2e-16 |
| **Gender** |  |  |  |  |  |  |  |  |  |  |
| **Female** | 25817 | 54.59 | Reference |  |  |  |  |  |  |  |
| **Male** | 21477 | 45.41 | 0.4407 | 1.55 | 1.39-1.74 | 1.38E-14 | 0.336315 | 1.4 | 1.25-1.57 | 1.04E-08 |
| **Age** |  |  |  |  |  |  |  |  |  |  |
| **40-59** | 21854 | 46.21 | Reference |  |  |  |  |  |  |  |
| **60-** | 25440 | 53.79 | 1.11314 | 3.04 | 2.67-3.48 | <2e-16 | 0.983566 | 2.67 | 2.34-3.07 | < 2e-16 |
| **Education** |  |  |  |  |  |  |  |  |  |  |
| **Elementary school above** | 35808 | 75.71 | Reference |  |  |  |  |  |  |  |
| **Elementary School/below** | 11486 | 24.29 | -0.08595 | 0.92 | 0.80-1.05 | 0.204 |  |  |  |  |
| **Occupation** |  |  |  |  |  |  |  |  |  |  |
| **mental work** | 13674 | 28.91 | Reference |  |  |  |  |  |  |  |
| **manual work** | 33620 | 71.09 | -0.007611 | 0.99 | 0.88-1.12 | 0.903 |  |  |  |  |
| **Residential area** |  |  |  |  |  |  |  |  |  |  |
| **central urban** | 17667 | 37.36 | Reference |  |  |  |  |  |  |  |
| **agriculture-related areas** | 29627 | 62.64 | -0.58536 | 0.56 | 0.50-0.62 | <2e-16 | -0.511221 | 0.6 | 0.53-0.67 | < 2e-16 |
| **FIT** |  |  |  |  |  |  |  |  |  |  |
| **Negative** | 15609 | 33.00 | Reference |  |  |  |  |  |  |  |
| **Positive** | 31685 | 67.00 | 0.94597 | 2.58 | 2.22-3.00 | <2e-16 | 1.151963 | 3.16 | 2.62-3.83 | < 2e-16 |
| **History of chronic diarrhea** |  |  |  |  |  |  |  |  |  |  |
| **No** | 36548 | 77.28 | Reference |  |  |  |  |  |  |  |
| **Yes** | 10746 | 22.72 | -0.46681 | 0.63 | 0.54-0.73 | 1.98E-09 | -0.137234 | 0.87 | 0.74-1.02 | 0.093 |
| **History of chronic constipation** |  |  |  |  |  |  |  |  |  |  |
| **No** | 36299 | 76.75 | Reference |  |  |  |  |  |  |  |
| **Yes** | 10995 | 23.25 | -0.47914 | 0.62 | 0.53-0.72 | 5.80E-10 | -0.253505 | 0.78 | 0.66-0.91 | 0.00174 |
| **History of bloody mucous stools** |  |  |  |  |  |  |  |  |  |  |
| **No** | 39478 | 83.47 | Reference |  |  |  |  |  |  |  |
| **Yes** | 7816 | 16.53 | 0.04397 | 1.04 | 0.90-1.21 | 0.56 |  |  |  |  |
| **History of chronic appendicitis or appendectomy** |  |  |  |  |  |  |  |  |  |  |
| **No** | 42574 | 90.02 | Reference |  |  |  |  |  |  |  |
| **Yes** | 4720 | 9.98 | -0.43529 | 0.65 | 0.52-0.80 | 0.000113 | -0.214303 | 0.81 | 0.64-1.01 | 0.06665 |
| **History of chronic cholecystitis or gallstones** |  |  |  |  |  |  |  |  |  |  |
| **No** | 42921 | 90.75 | Reference |  |  |  |  |  |  |  |
| **Yes** | 4373 | 9.25 | -0.2166 | 0.81 | 0.65-0.99 | 0.0426 | -0.009592 | 0.99 | 0.79-1.23 | 0.93231 |
| **Adverse life events** |  |  |  |  |  |  |  |  |  |  |
| **No** | 41659 | 88.09 | Reference |  |  |  |  |  |  |  |
| **Yes** | 5635 | 11.91 | -0.24997 | 0.78 | 0.64-0.94 | 0.00943 | 0.06687 | 1.07 | 0.87-1.30 | 0.51164 |
| **History of cancer** |  |  |  |  |  |  |  |  |  |  |
| **No** | 45743 | 96.72 | Reference |  |  |  |  |  |  |  |
| **Yes** | 1551 | 3.28 | -0.2168 | 0.81 | 0.56-1.12 | 0.217 |  |  |  |  |
| **History of CRC in a first-degree relative** |  |  |  |  |  |  |  |  |  |  |
| **No** | 42351 | 89.55 | Reference |  |  |  |  |  |  |  |
| **Yes** | 4943 | 10.45 | -0.48769 | 0.61 | 0.49-0.76 | 1.50E-05 | -0.149259 | 0.86 | 0.67-1.09 | 0.22411 |

NOTE: The number of participants in this logistic regression was 47294.

**③Reference group: ＜3 months**

|  |  | | **Unadjusted** | | | | **Adjusted** | | | |
| --- | --- | --- | --- | --- | --- | --- | --- | --- | --- | --- |
|  | **n** | **%** | **Beta** | **OR** | **95%CI** | **p Value** | **Beta** | **OR** | **95%CI** | **p Value** |
| **Time to Colonoscopy after FIT, mo** |  |  |  |  |  |  |  |  |  |  |
| **＜3** | 33835 | 67.93 | Reference |  |  |  |  |  |  |  |
| **3-4** | 3048 | 6.12 | 0.0007748 | 1 | 0.77-1.28 | 0.995 | 0.016014 | 1.02 | 0.78-1.31 | 0.90355 |
| **4-5** | 1701 | 3.41 | -0.0357605 | 0.96 | 0.67-1.34 | 0.838 | -0.004358 | 1 | 0.69-1.38 | 0.98027 |
| **5-6** | 1046 | 2.10 | 0.1963321 | 1.22 | 0.81-1.76 | 0.323 | 0.184247 | 1.2 | 0.79-1.74 | 0.35739 |
| **6-12** | 2453 | 4.92 | 0.5475983 | 1.73 | 1.37-2.15 | 1.68E-06 | 0.637629 | 1.89 | 1.50-2.36 | 4.41E-08 |
| **12-24** | 2394 | 4.81 | 0.7250576 | 2.06 | 1.66-2.54 | 1.55E-11 | 0.958461 | 2.61 | 2.09-3.22 | < 2e-16 |
| **24-36** | 1739 | 3.49 | 0.9189576 | 2.51 | 1.99-3.12 | 1.07E-15 | 1.190327 | 3.29 | 2.59-4.12 | < 2e-16 |
| **>36** | 3594 | 7.22 | 0.9849858 | 2.68 | 2.27-3.14 | < 2e-16 | 1.368389 | 3.93 | 3.31-4.65 | < 2e-16 |
| **Gender** |  |  |  |  |  |  |  |  |  |  |
| **Female** | 27155 | 54.52 | Reference |  |  |  |  |  |  |  |
| **Male** | 22655 | 45.48 | 0.43811 | 1.55 | 1.39-1.73 | 5.38E-15 | 0.336101 | 1.4 | 1.25-1.57 | 4.97E-09 |
| **Age** |  |  |  |  |  |  |  |  |  |  |
| **40-59** | 22898 | 45.97 | Reference |  |  |  |  |  |  |  |
| **60-** | 26912 | 54.03 | 1.09582 | 2.99 | 2.63-3.41 | <2e-16 | 0.948208 | 2.58 | 2.26-2.95 | < 2e-16 |
| **Education** |  |  |  |  |  |  |  |  |  |  |
| **Elementary school above** | 37648 | 75.58 | Reference |  |  |  |  |  |  |  |
| **Elementary School/below** | 12162 | 24.42 | -0.08237 | 0.92 | 0.81-1.05 | 0.213 |  |  |  |  |
| **Occupation** |  |  |  |  |  |  |  |  |  |  |
| **mental work** | 14418 | 28.95 | Reference |  |  |  |  |  |  |  |
| **manual work** | 35392 | 71.05 | 0.02041 | 1.02 | 0.91-1.15 | 0.74 |  |  |  |  |
| **Residential area** |  |  |  |  |  |  |  |  |  |  |
| **central urban** | 18524 | 37.19 | Reference |  |  |  |  |  |  |  |
| **agriculture-related areas** | 31286 | 62.81 | -0.58878 | 0.56 | 0.50-0.62 | <2e-16 | -0.523267 | 0.59 | 0.53-0.66 | < 2e-16 |
| **FIT** |  |  |  |  |  |  |  |  |  |  |
| **Negative** | 16341 | 32.81 | Reference |  |  |  |  |  |  |  |
| **Positive** | 33469 | 67.19 | 0.94218 | 2.57 | 2.22-2.98 | <2e-16 | 1.156141 | 3.18 | 2.64-3.83 | < 2e-16 |
| **History of chronic diarrhea** |  |  |  |  |  |  |  |  |  |  |
| **No** | 38490 | 77.27 | Reference |  |  |  |  |  |  |  |
| **Yes** | 11320 | 22.73 | -0.42486 | 0.65 | 0.56-0.76 | 1.56E-08 | -0.092518 | 0.91 | 0.78-1.06 | 0.24111 |
| **History of chronic constipation** |  |  |  |  |  |  |  |  |  |  |
| **No** | 38242 | 76.78 | Reference |  |  |  |  |  |  |  |
| **Yes** | 11568 | 23.22 | -0.48209 | 0.62 | 0.53-0.71 | 2.04E-10 | -0.250857 | 0.78 | 0.66-0.91 | 0.00156 |
| **History of bloody mucous stools** |  |  |  |  |  |  |  |  |  |  |
| **No** | 41530 | 83.38 | Reference |  |  |  |  |  |  |  |
| **Yes** | 8280 | 16.62 | 0.0882 | 1.09 | 0.95-1.26 | 0.225 |  |  |  |  |
| **History of chronic appendicitis or appendectomy** |  |  |  |  |  |  |  |  |  |  |
| **No** | 44890 | 90.12 | Reference |  |  |  |  |  |  |  |
| **Yes** | 4820 | 9.68 | -0.40795 | 0.67 | 0.53-0.82 | 0.000199 | -0.191095 | 0.83 | 0.66-1.03 | 0.09268 |
| **History of chronic cholecystitis or gallstones** |  |  |  |  |  |  |  |  |  |  |
| **No** | 45239 | 90.82 | Reference |  |  |  |  |  |  |  |
| **Yes** | 4571 | 9.18 | -0.22114 | 0.8 | 0.65-0.98 | 0.0355 | -0.017867 | 0.98 | 0.79-1.21 | 0.87213 |
| **Adverse life events** |  |  |  |  |  |  |  |  |  |  |
| **No** | 43951 | 88.24 | Reference |  |  |  |  |  |  |  |
| **Yes** | 5859 | 11.76 | -0.24635 | 0.78 | 0.65-0.94 | 0.00927 | 0.059634 | 1.06 | 0.87-1.29 | 0.55097 |
| **History of cancer** |  |  |  |  |  |  |  |  |  |  |
| **No** | 48189 | 96.75 | Reference |  |  |  |  |  |  |  |
| **Yes** | 1621 | 3.25 | -0.22266 | 0.8 | 0.56-1.11 | 0.199 |  |  |  |  |
| **History of CRC in a first-degree relative** |  |  |  |  |  |  |  |  |  |  |
| **No** | 44669 | 89.68 | Reference |  |  |  |  |  |  |  |
| **Yes** | 5141 | 10.32 | -0.50684 | 0.6 | 0.48-0.75 | 5.89E-06 | -0.168873 | 0.84 | 0.66-1.07 | 0.16462 |

NOTE: The number of participants in this logistic regression was 49810.

**④Reference group: ＜6 months**

|  |  | | **Unadjusted** | | | | **Adjusted** | | | |
| --- | --- | --- | --- | --- | --- | --- | --- | --- | --- | --- |
|  | **n** | **%** | **Beta** | **OR** | **95%CI** | **p Value** | **Beta** | **OR** | **95%CI** | **p Value** |
| **Time to Colonoscopy after FIT, mo** |  |  |  |  |  |  |  |  |  |  |
| **＜6** | 39630 | 79.56 | Reference |  |  |  |  |  |  |  |
| **6-12** | 2453 | 4.92 | 0.54336 | 1.72 | 1.37-2.14 | 1.66E-06 | 0.63082 | 1.88 | 1.49-2.34 | 4.66E-08 |
| **12-24** | 2394 | 4.81 | 0.72082 | 2.06 | 1.66-2.52 | 1.33E-11 | 0.95165 | 2.59 | 2.08-3.19 | < 2e-16 |
| **24-36** | 1739 | 3.49 | 0.91472 | 2.5 | 1.98-3.10 | 8.51E-16 | 1.18337 | 3.27 | 2.58-4.09 | < 2e-16 |
| **>36** | 3594 | 7.22 | 0.98074 | 2.67 | 2.27-3.12 | < 2e-16 | 1.36146 | 3.9 | 3.29-4.61 | < 2e-16 |
| **Gender** |  |  |  |  |  |  |  |  |  |  |
| **Female** | 27155 | 54.52 | Reference |  |  |  |  |  |  |  |
| **Male** | 22655 | 45.48 | 0.43811 | 1.55 | 1.39-1.73 | 5.38E-15 | 0.33588 | 1.4 | 1.25-1.57 | 5.07E-09 |
| **Age** |  |  |  |  |  |  |  |  |  |  |
| **40-59** | 22898 | 45.97 | Reference |  |  |  |  |  |  |  |
| **60-** | 26912 | 54.03 | 1.09582 | 2.99 | 2.63-3.41 | <2e-16 | 0.95043 | 2.59 | 2.27-2.96 | < 2e-16 |
| **Education** |  |  |  |  |  |  |  |  |  |  |
| **Elementary school above** | 37648 | 75.58 | Reference |  |  |  |  |  |  |  |
| **Elementary School/below** | 12162 | 24.42 | -0.08237 | 0.92 | 0.81-1.05 | 0.213 |  |  |  |  |
| **Occupation** |  |  |  |  |  |  |  |  |  |  |
| **mental work** | 14418 | 28.95 | Reference |  |  |  |  |  |  |  |
| **manual work** | 35392 | 71.05 | 0.02041 | 1.02 | 0.91-1.15 | 0.74 |  |  |  |  |
| **Residential area** |  |  |  |  |  |  |  |  |  |  |
| **central urban** | 18524 | 37.19 | Reference |  |  |  |  |  |  |  |
| **agriculture-related areas** | 31286 | 62.81 | -0.58878 | 0.56 | 0.50-0.62 | <2e-16 | -0.52313 | 0.59 | 0.53-0.66 | < 2e-16 |
| **FIT** |  |  |  |  |  |  |  |  |  |  |
| **Negative** | 16341 | 32.81 | Reference |  |  |  |  |  |  |  |
| **Positive** | 33469 | 67.19 | 0.94218 | 2.57 | 2.22-2.98 | <2e-16 | 1.15412 | 3.17 | 2.64-3.82 | < 2e-16 |
| **History of chronic diarrhea** |  |  |  |  |  |  |  |  |  |  |
| **No** | 38490 | 77.27 | Reference |  |  |  |  |  |  |  |
| **Yes** | 11320 | 22.73 | -0.42486 | 0.65 | 0.56-0.76 | 1.56E-08 | -0.09302 | 0.91 | 0.78-1.06 | 0.2385 |
| **History of chronic constipation** |  |  |  |  |  |  |  |  |  |  |
| **No** | 38242 | 76.78 | Reference |  |  |  |  |  |  |  |
| **Yes** | 11568 | 23.22 | -0.48209 | 0.62 | 0.53-0.71 | 2.04E-10 | -0.25079 | 0.78 | 0.66-0.91 | 0.00157 |
| **History of bloody mucous stools** |  |  |  |  |  |  |  |  |  |  |
| **No** | 41530 | 83.38 | Reference |  |  |  |  |  |  |  |
| **Yes** | 8280 | 16.62 | 0.0882 | 1.09 | 0.95-1.26 | 0.225 |  |  |  |  |
| **History of chronic appendicitis or appendectomy** |  |  |  |  |  |  |  |  |  |  |
| **No** | 44890 | 90.12 | Reference |  |  |  |  |  |  |  |
| **Yes** | 4820 | 9.68 | -0.40795 | 0.67 | 0.53-0.82 | 0.000199 | -0.19112 | 0.83 | 0.66-1.03 | 0.09259 |
| **History of chronic cholecystitis or gallstones** |  |  |  |  |  |  |  |  |  |  |
| **No** | 45239 | 90.82 | Reference |  |  |  |  |  |  |  |
| **Yes** | 4571 | 9.18 | -0.22114 | 0.8 | 0.65-0.98 | 0.0355 | -0.01819 | 0.98 | 0.79-1.21 | 0.86987 |
| **Adverse life events** |  |  |  |  |  |  |  |  |  |  |
| **No** | 43951 | 88.24 | Reference |  |  |  |  |  |  |  |
| **Yes** | 5859 | 11.76 | -0.24635 | 0.78 | 0.65-0.94 | 0.00927 | 0.05947 | 1.06 | 0.87-1.29 | 0.55185 |
| **History of cancer** |  |  |  |  |  |  |  |  |  |  |
| **No** | 48189 | 96.75 | Reference |  |  |  |  |  |  |  |
| **Yes** | 1621 | 3.25 | -0.22266 | 0.8 | 0.56-1.11 | 0.199 |  |  |  |  |
| **History of CRC in a first-degree relative** |  |  |  |  |  |  |  |  |  |  |
| **No** | 44669 | 89.68 | Reference |  |  |  |  |  |  |  |
| **Yes** | 5141 | 10.32 | -0.50684 | 0.6 | 0.48-0.75 | 5.89E-06 | -0.1683 | 0.85 | 0.66-1.07 | 0.16605 |

NOTE: The number of participants in this logistic regression was 49810.

**⑤Reference group: ＜1 months, and includes who had polyp before FIT screening.**

|  |  | | **Unadjusted** | | | | **Adjusted** | | | |
| --- | --- | --- | --- | --- | --- | --- | --- | --- | --- | --- |
|  | **n** | **%** | **Beta** | **OR** | **95%CI** | **p Value** | **Beta** | **OR** | **95%CI** | **p Value** |
| **Time to Colonoscopy after FIT, mo** |  |  |  |  |  |  |  |  |  |  |
| **＜1** | 21249 | 39.30 | Reference |  |  |  |  |  |  |  |
| **1-2** | 9305 | 17.21 | -0.23201 | 0.79 | 0.66-0.95 | 0.01102 | -0.27654 | 0.76 | 0.63-0.91 | 0.002645 |
| **2-3** | 5821 | 10.76 | -0.24412 | 0.78 | 0.63-0.97 | 0.02671 | -0.29692 | 0.74 | 0.59-0.92 | 0.007585 |
| **3-4** | 3292 | 6.09 | -0.10689 | 0.9 | 0.69-1.16 | 0.42072 | -0.11447 | 0.89 | 0.68-1.15 | 0.39378 |
| **4-5** | 1850 | 3.42 | -0.13731 | 0.87 | 0.61-1.21 | 0.43162 | -0.12209 | 0.89 | 0.62-1.23 | 0.488131 |
| **5-6** | 1153 | 2.13 | 0.08912 | 1.09 | 0.73-1.58 | 0.65079 | 0.07579 | 1.08 | 0.71-1.56 | 0.702922 |
| **6-12** | 2819 | 5.21 | 0.38199 | 1.47 | 1.16-1.83 | 0.00102 | 0.4929 | 1.64 | 1.29-2.06 | 3.29E-05 |
| **12-24** | 2744 | 5.07 | 0.55826 | 1.75 | 1.40-2.16 | 3.73E-07 | 0.80734 | 2.24 | 1.79-2.79 | 8.17E-13 |
| **24-36** | 1952 | 3.61 | 0.7985 | 2.22 | 1.76-2.77 | 4.72E-12 | 1.06602 | 2.9 | 2.29-3.65 | < 2e-16 |
| **>36** | 3890 | 7.19 | 0.88828 | 2.43 | 2.05-2.87 | < 2e-16 | 1.24037 | 3.46 | 2.89-4.12 | < 2e-16 |
| **Gender** |  |  |  |  |  |  |  |  |  |  |
| **Female** | 29128 | 53.87 | Reference |  |  |  |  |  |  |  |
| **Male** | 24947 | 46.13 | 0.40687 | 1.5 | 1.35-1.67 | 1.77E-13 | 0.32693 | 1.39 | 1.24-1.55 | 7.90E-09 |
| **Age** |  |  |  |  |  |  |  |  |  |  |
| **40-59** | 24542 | 45.39 | Reference |  |  |  |  |  |  |  |
| **60-** | 29533 | 54.61 | 1.0538 | 2.87 | 2.53-3.26 | <2e-16 | 0.95183 | 2.59 | 2.28-2.96 | < 2e-16 |
| **Education** |  |  |  |  |  |  |  |  |  |  |
| **Elementary school above** | 41260 | 76.30 | Reference |  |  |  |  |  |  |  |
| **Elementary School/below** | 12815 | 23.70 | -0.03461 | 0.97 | 0.85-1.10 | 0.595 |  |  |  |  |
| **Occupation** |  |  |  |  |  |  |  |  |  |  |
| **mental work** | 15790 | 29.20 | Reference |  |  |  |  |  |  |  |
| **manual work** | 38285 | 70.80 | 0.03772 | 1.04 | 0.92-1.17 | 0.535 |  |  |  |  |
| **Residential area** |  |  |  |  |  |  |  |  |  |  |
| **central urban** | 20628 | 38.15 | Reference |  |  |  |  |  |  |  |
| **agriculture-related areas** | 33447 | 61.85 | -0.54455 | 0.58 | 0.52-0.65 | <2e-16 | -0.50718 | 0.6 | 0.54-0.67 | < 2e-16 |
| **FIT** |  |  |  |  |  |  |  |  |  |  |
| **Negative** | 19556 | 36.16 | Reference |  |  |  |  |  |  |  |
| **Positive** | 34519 | 63.84 | 0.97612 | 2.65 | 2.31-3.06 | <2e-16 | 1.07285 | 2.92 | 2.45-3.49 | < 2e-16 |
| **History of chronic diarrhea** |  |  |  |  |  |  |  |  |  |  |
| **No** | 41769 | 77.24 | Reference |  |  |  |  |  |  |  |
| **Yes** | 12306 | 22.76 | -0.41437 | 0.66 | 0.57-0.76 | 1.98E-08 | -0.10593 | 0.9 | 0.77-1.04 | 0.170955 |
| **History of chronic constipation** |  |  |  |  |  |  |  |  |  |  |
| **No** | 41638 | 77.00 | Reference |  |  |  |  |  |  |  |
| **Yes** | 12437 | 23.00 | -0.46143 | 0.63 | 0.54-0.73 | 6.19E-10 | -0.25969 | 0.77 | 0.66-0.90 | 0.000857 |
| **History of bloody mucous stools** |  |  |  |  |  |  |  |  |  |  |
| **No** | 45201 | 83.59 | Reference |  |  |  |  |  |  |  |
| **Yes** | 8874 | 16.41 | 0.09141 | 1.1 | 0.95-1.26 | 0.204 |  |  |  |  |
| **History of chronic appendicitis or appendectomy** |  |  |  |  |  |  |  |  |  |  |
| **No** | 48787 | 90.22 | Reference |  |  |  |  |  |  |  |
| **Yes** | 5288 | 9.78 | -0.40159 | 0.67 | 0.54-0.82 | 0.000212 | -0.2088 | 0.81 | 0.65-1.01 | 0.062724 |
| **History of chronic cholecystitis or gallstones** |  |  |  |  |  |  |  |  |  |  |
| **No** | 49159 | 90.91 | Reference |  |  |  |  |  |  |  |
| **Yes** | 4916 | 9.09 | -0.21796 | 0.8 | 0.65-0.98 | 0.0362 | -0.04091 | 0.96 | 0.77-1.18 | 0.708764 |
| **Adverse life events** |  |  |  |  |  |  |  |  |  |  |
| **No** | 47883 | 88.55 | Reference |  |  |  |  |  |  |  |
| **Yes** | 6192 | 11.45 | -0.2451 | 0.78 | 0.65-0.94 | 0.00949 | 0.02206 | 1.02 | 0.84-1.24 | 0.82459 |
| **History of cancer** |  |  |  |  |  |  |  |  |  |  |
| **No** | 52378 | 96.86 | Reference |  |  |  |  |  |  |  |
| **Yes** | 1697 | 3.14 | -0.1825 | 0.83 | 0.59-1.15 | 0.285 |  |  |  |  |
| **History of polyp** |  |  |  |  |  |  |  |  |  |  |
| **No** | 49810 | 92.11 | Reference |  |  |  |  |  |  |  |
| **Yes** | 4265 | 7.89 | -1.16868 | 0.31 | 0.22-0.43 | 5.65E-12 | -0.95398 | 0.39 | 0.27-0.53 | 4.44E-08 |
| **History of CRC in a first-degree relative** |  |  |  |  |  |  |  |  |  |  |
| **No** | 48623 | 89.92 | Reference |  |  |  |  |  |  |  |
| **Yes** | 5452 | 10.08 | -0.48406 | 0.62 | 0.49-0.76 | 1.20E-05 | -0.20157 | 0.82 | 0.64-1.03 | 0.091552 |

NOTE: The number of participants in this logistic regression was 54075.
